# SARS-CoV-2 and COVID-19: A Narrative Review

**DOI:** 10.3389/bjbs.2022.10426

**Published:** 2022-09-06

**Authors:** A. D. Blann, R. Heitmar

**Affiliations:** School of Applied Sciences, University of Huddersfield Queensgate, Huddersfield, United Kingdom

**Keywords:** COVID-19, SARS-CoV-2, vaccination, variants, long COVID

## Abstract

The World Health Organisation has reported that the viral disease known as COVID-19, caused by SARS-CoV-2, is the leading cause of death by a single infectious agent. This narrative review examines certain components of the pandemic: its origins, early clinical data, global and UK-focussed epidemiology, vaccination, variants, and long COVID.

## Introduction

To say that the COVID-19 pandemic is the first major global health issue for a little over a century is to state the obvious. The continuously rolling stages of the pandemic, and our responses to it, have for the time being made it difficult to draw firm conclusions, and any long-term effects will not be known for years to come. In the early stages of the third year of the pandemic, the present review will build on, and update, a previous review in this Journal (1) and provide an update on the state of the pandemic globally and in the United Kingdom (UK).

## The Origins of SARS-CoV-2

Much of the history of the origins of this virus is known (1–3). On 12 December 2019, health authorities in the city of Wuhan in the Hubei region of China reported an unusual idiopathic pneumonia, some cases progressing to respiratory failure, which is the major criterion of an acute respiratory disease syndrome (ARDS) (4). Six of the first seven patients worked at a local seafood market, and samples were sent to the local laboratory, which identified a candidate pathogen as a coronavirus, named 2019-nCoV. Subsequent analysis showed a 79.6%–87.1% sequence matching with known SARS-CoV viruses MERS-CoV and SARS-COV, whose intermediate hosts are the dromedary and civet cat, respectively). Later, a >95% overall genome sequence identity likeness with the virus BatVoVRaTG13, previously detected in the bat *Rhinolophus affinis*, was reported, suggesting a common ancestor (4, 5). Consequently, this phylogenetic relationship provided evidence that the virus may have originated in bats, though alternative hosts such as civet cats and pangolins were suggested, a SARS-CoV-like virus being isolated from the latter (4–6). Genome analysis pointed to an envelope spike protein interacting with angiotensin-converting enzyme 2 (ACE2) on the target cell membrane as the entry point. The sequence of the virus was determined and published early in 2020 (6–9), and it was subsequently re-named SARS-Cov-2 in March 2020 (10).

The first official [as per the World Health Organisation (WHO)] case was confirmed on 30 December 2019 and the first death on 6 January 2020. Data were regularly forwarded to the WHO, who, on 20 January 2020, began publishing weekly Situation Reports (11). The 23rd Report, dated 12 February, referred to the disease as COVID-19 (12).

## Early Clinical and Laboratory Data From China

Apart from the atypical pneumonia, a principle early observation regarded the infection rate and its high transmissibility, with a reproduction rate (R) of 2.5, to be considerably higher than influenza (R = 0.93) and other coronaviruses MERS-CoV (R = 0.69) and SARS-Cov-1 (R = 1.1) (13). Other data followed, pointing to leading symptoms typical of a severe viral infection of the lungs, i.e., fever (83%), cough (82%), and shortness of breath (31%), with 15% of patients reporting all three, whilst over half had a chronic medical illness (notably hypertension, diabetes, and cerebrovascular disease), and there was a case fatality rate of 11% (14). A subsequent report (15) showed that leading risk factors for admission to an intensive care unit (ICU) were age, hypertension, cerebrovascular disease, diabetes, dyspnea, and dizziness (all *p* < 0.01). Leading laboratory findings linked to ICU admission were increased neutrophil count, D-dimers, creatine kinase, lactate dehydrogenase, urea, aspartate aminotransferase, high-sensitivity troponin I, and procalcitonin (all *p* < 0.01). Invasive mechanical ventilation was required by 47% of those admitted to ICU. A further study found that age, troponin I, lactate dehydrogenase, and creatine kinase were all independent predictors of mortality (16). These data were subsequently confirmed and extended in Caucasian populations (see sections to come).

## The Epidemiology of the Developing Pandemic

### Global Perspectives

A year after the initial cases (12 December 2019), the disease had become global, present in all populated continents, with around 76.2 million people infected (0.97% of the world’s population), causing 1.8 million deaths (2.3%) of those infected. The WHO coronavirus (COVID-19) dashboard continued to provide two major metrics: the numbers of infected people (cases) and the number of deaths (2). Such data showing the increase in the rates of cases and death, and thus any changes in the rates of death per case, up to December 2021 are presented in Table 1.

**TABLE 1:** Global COVID-19 cases and deaths.

Month*	2020			2021		
	Cases	Deaths	Percentage of deaths	Cases	Deaths	Percentage of deaths
January	**	**	**	89.6	1.99	2.22
February	0.047	0.001	2.70	105.6	2.39	2.26
March	0.11	0.036	3.36	116.2	2.67	2.30
April	1.18	0.066	5.59	134.9	3.00	2.22
May	3.96	0.285	7.20	157.1	3.39	2.16
June	6.79	0.417	6.14	175.4	3.75	2.14
July	12.6	0.550	4.36	186.3	4.00	2.15
August	19.6	0.763	3.89	202.3	4.28	2.11
September	29.1	0.970	3.33	224.3	4.62	2.06
October	37.9	1.166	3.08	237.1	4.84	2.04
November	50.9	1.311	2.58	246.7	5.00	2.03
December	71.7	1.670	2.33	262.3	5.22	1.99

Raw data: millions. *First of each month. **Numbers too small to be reliable. In early 2022, these data increased further to 324.31, 5.55 and 1.7%, respectively in January, to 411.86, 5.84, and 1.4% in February, to 459.1, 6.07, and 1.3% in March, and to 501.14, 6.2, and 1.2 in April. In going to press on 6 May 2022, data were 513.95, 6.25, and 1.2%, respectively.

**FIGURE 1:**
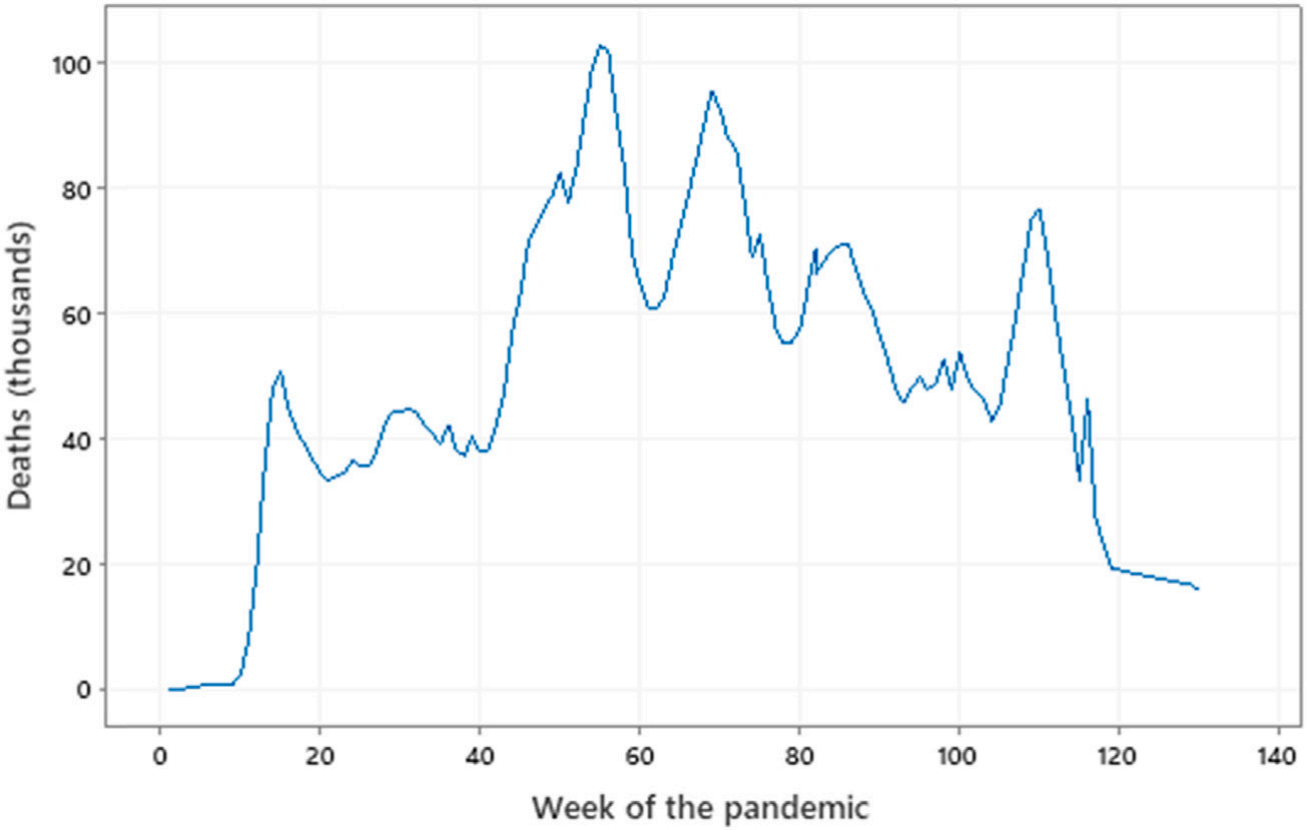
Global deaths throughout the pandemic. Data from the WHO (covid19.who-int). Week 0 = 6 January 2020, Week 60 = 22 February 2021, Week 120 = 18 April 2021. Several peaks and troughs are evident.

Whilst there were a number of peaks and troughs, broadly speaking, the number of deaths increased in an approximately straight line during 2020 and 2021, and although the number of cases also increased in an approximately straight line, the rate increased markedly in early 2022. The unusual early peak in the proportion of deaths to cases (as high as 7.2% in May 2020, Table 1) may be explained in terms of the early difficulties in recognising and defining both a case and a death. Lack of consensus on a definition of a case in the absence of a scientifically proven infection is a potential reason, and so many cases and deaths may have been assumed to be due to COVID-19. Thus, understanding of the spread of the virus and its effects was hampered by the non-standard definitions of infection and related deaths and lack of an accurate and accessible test for either the virus itself or proof of infection (i.e., the presence of serum antibodies). Once the data collection had settled down after the first 6 months, several clear peaks in both number of cases and number deaths are evident, though the precise reason(s) for this are unclear (Figure 1).

Many nations were slow to come to terms with the virus and its effects. Some promptly placed travel barriers to effectively ban migration and other restrictions (such as the closure of schools and universities and implementation of night-time curfews), whilst others took longer to adopt the same interventions. One of the greatest disparities is presumably due to the frequency of testing, how cases and deaths are reported, countries not releasing data (e.g., China), etc. A second, independent factor was the ability of nations, and their health care systems, to respond to the infections.

These factors may be reflected by the data in Table 2, which compares the cumulative increases in the number of confirmed cases some 6 months after the pandemic started, at 1 year and at 18 months in selected nations. This data is also adjusted for the population of each nation and so points to great variation in the relative spread of the disease between nations, regions, and continents. Similarly, Table 3 shows parallel data of the increases in case-related fatalities, also adjusted for the size of the population. **I**nterpretation of these data in the initial 6 months to mid-June 2020 is problematical because of, as described above, the likelihood of different methods used to define a case and a death (e.g., in hospitals only or in hospitals and elsewhere), known to vary between countries, over time. As the pandemic developed, objective clinical definitions and the wider availability of scientific testing provided more reliable data.

**TABLE 2:** Data on COVID-19 confirmed cases from selected nations.

Nation	Mid-June 2020		Mid-December 2020		Mid-June 2021	
	Confirmed cases (10^6^)	Confirmed cases % of population	Confirmed cases (10^6^)	Confirmed cases % of population	Confirmed cases (10^6^)	Confirmed cases % of population
The world	8.71	0.11	76.2	0.97	177.9	2.26
Argentina	0.04	0.09	1.48	3.32	4.24	9.52
Australia	<0.1	<0.01	0.03	0.11	0.03	0.11
Brazil	0.38	0.12	6.78	2.07	17.8	5.44
China	0.08	<0.01	0.09	<0.01	0.12	<0.01
Columbia	0.06	0.12	1.40	2.82	3.89	7.84
France	0.16	0.24	2.31	3.45	5.65	8.43
Germany	0.19	0.23	1.49	1.78	3.72	4.44
India	0.41	0.03	10.0	0.72	29.9	2.21
Italy	0.24	0.40	1.93	2.98	4.24	7.02
Mexico	0.17	0.13	1.30	1.03	2.47	1.96
Peru	0.27	0.84	0.99	3.09	2.02	6.31
Russia	0.58	0.40	2.85	1.82	5.32	3.68
Spain	0.25	0.53	1.83	3.75	3.77	8.04
South Africa	0.09	0.16	0.91	1.58	1.81	3.13
Sweden	0.05	0.50	0.34	3.32	1.08	10.6
United Kingdom	0.28	0.42	2.08	2.77	4.62	6.97
United States	2.22	0.68	17.5	5.35	33.2	10.1

All data from www.covid19.who.int

**TABLE 3:** Data on COVID-19 deaths from selected nations.

Nation	Mid-June 2020		Mid-December 2020		Mid-June 2021	
	Deaths (10^3^)	Deaths (% of population)	Deaths (10^3^)	Deaths (% of population)	Deaths (10^3^)	Deaths (% of population)
The world	486.2	<0.01	1752.7	0.02	3860.8	0.05
Argentina	0.98	<0.01	41.7	0.09	88.2	0.20
Australia	0.1	<0.01	0.9	<0.01	0.9	<0.01
Brazil	48.9	0.01	185.6	0.06	498.5	0.15
China	4.6	<0.01	4.8	<0.01	5.4	<0.01
Columbia	1.5	<0.01	40.0	0.08	98.7	0.20
France	29.5	0.04	60.0	0.09	109.8	0.16
Germany	8.9	0.01	26.0	0.03	90.4	0.11
India	13.3	<0.01	145.5	0.01	386.7	0.03
Italy	34.6	0.06	68.4	0.11	127.3	0.21
Mexico	20.4	0.02	117.2	0.09	230.9	0.18
Peru	32.0	0.10	91.6	0.29	189.9	0.59
Russia	8.1	0.01	50.8	0.03	129.4	0.09
Spain	29.7	0.06	52.1	0.11	81.1	0.17
South Africa	1.87	<0.01	24.5	0.04	59.8	0.10
UK	39.8	0.06	67.1	0.10	128.0	0.19
United States of America	120.1	0.04	319.0	0.10	596.1	0.18

All data from www.covid19.who.int

### Migration of the Virus to Europe

The daily situation reports from the WHO showed the slow but steady arrival of the virus in Europe in late January/early February 2020. Two Chinese visitors to Italy flew into Milan from Wuhan on 23 January 2020. Italy reported its first two patients on 29 January, precipitating the Italian epidemic and thus also the first hard data of the infection in Europe. Data from 2,619 Italians with proven COVID-19 (17) pointed to high frequencies of co-morbidities of hypertension (68.5%), type 2 diabetes (30.9%), ischaemic heart disease (28.7%), atrial fibrillation (22.3% and chronic renal failure (20.4). Only 3.8% of patients failed to have a co-morbidity. Leading symptoms were fever (76.2%), dyspnoea (73.5) and cough (38.5%), with 20.3% requiring intensive care. A subsequent report (18) of 4551 symptomatic subjects found 58.3% to be infected, of whom 40% were hospitalised and of whom 20.2% died (8.2% of the infected cohort). Leading co-morbidities in those admitted were as in the work of Canveli et al. (17), to which dyslipidaemia (72.0%), chronic obstructive pulmonary disease (COPD, 71.1%), heart failure (70.1%), cancer (55.5%), obesity (52.3%) and dementia (46.7%) were added. However, of these, coronary heart disease, dementia, diabetes, hypertension, heart failure, cancer, and arrhythmia were significant predictors of death.

The index case for the epidemic in Spain was not identified, the first two confirmed cases being reported on 27 January 2020. Subsequently, infections, hospitalisations, and death (250,000 cases and 25,000 deaths within 8 weeks) proportionately exceeded those of Italy. Epidemiological audits were broadly similar to those from Italy, but in addition, factors measured 72 h after admission to ICU and that predicted death included poor oxygenation, increased white cells and neutrophils, creatinine, urea, lactate dehydrogenase, procalcitonin, D-dimers and a reduced lymphocyte count (19). Notably, these data from Italy and Spain effectively confirmed those from China (14–16). Random sampling from over 35,000 Spanish households indicated an infection rate of 1 in 20 (20). The adverse course of the epidemic in Spain was ascribed to a disproportionate loss of health care workers, shortage of diagnostic tests and personal protective equipment, and a late social lockdown, among other factors (21).

### Appearance of the Virus in the United Kingdom

Before Wuhan was put into lockdown and the airport closed on 23 January, 17 direct passenger flights had already landed in the UK and over 600 from the whole of China. The UK’s “Patient Zero” was likely to be two individuals, also from China, and diagnosed on clinical grounds on 27 February 2020. It was also noted that many UK residents returned from holiday in northern Italy, and so may well have brought the virus with them. As borders were open, and with many UK residents visiting Spain, it is also likely that this nation was an additional source. On 11 March, Liverpool Football Club hosted 3,000 visiting fans from Madrid: a week later the Government announced the school closures, and on 26 March, lockdown measures came into force.

Table 4 shows data on the number of cases and case fatality in certain European nations at a key milestone of the pandemic, the middle of June 2020, being 6 months from the initial description of the disease (1–4). These data underline considerable variation in national responses to the disease, from both the authorities and the populations themselves. Since the populations of these nations are genetically and demographically broadly equivalent, it follows that differences must be due to data collection, and the policies and practices of each nation, such as the mandatory use of face masks, social distancing, self-isolation when symptomatic, and the closure of schools and other establishments (22–28).

**TABLE 4:** Cases and deaths in Europe by mid-June 2020 of the first wave.

Nation	Cases	Deaths	% Deaths of cases
France	230	44	19.0
Germany	225	11	4.7
Italy	390	57	14.5
Spain	525	63	12.0
Sweden	504	50	9.9
The UK	444	63	14.2

Data adjusted for 100,000 of the population.

## The Pathology of COVID-19

### The Lung

The leading pathology reported from Wuhan was an atypical pneumonia, confirmed in Europeans, which in many cases deteriorated to acute respiratory distress syndrome (ARDS) with hypoxia (14–19). In the face of a potential pneumonia (or, indeed, any such pulmonary condition), lung function may be assessed by peripheral blood oxygenation, the most simple being the transdermal oxygenation sensor that fits on a finger. Ideally, this would give a result ≥95%, levels below this implying poor pulmonary oxygen uptake. A more precise method provides the partial pressure of blood oxygen (pO_2_), and its ratio with the fractional inspired oxygen (FiO_2_), hence pO_2_/FiO_2_. A ratio of 400–500 mm Hg is expected in healthy individuals and falls with increasing severity of acute respiratory disease. This is likely to be important in assessing those that may require mechanical ventilation and/or intensive care.

X-rays provide limited information (e.g., shadowing), although CT and MRI are more helpful in defining pulmonary lesions, the leading sign being “ground glass opacity,” present in 20–60% of cases (29). Hypoxia, reflecting poor lung function, is a risk factor for admission to intensive care and death (30), whilst existing interstitial lung fibrosis (ILF) before a COVID-19 infection brings a hazard ratio (HR)[95% confidence interval (CI)] of 1.6 (1.17–2.18) for survival, rising to 1.72 (1.05–2.83) in those with a forced vital capacity ≤80%, and to 2.27 (1.9–3.71) in those with obesity and ILF (31). A long-term consequence of acute lung damage is irreversible ILF (32), and this is a likely component of long Covid (33).

### Inflammation

The leading aetiology of pneumonia is local inflammation, impossible to confirm in the absence of an alveoli biopsy, although lower lung washing can provide clues (e.g., the presence of leukocytes, which may also contain a potentially pathognomic microorganism that may be causing pneumonia) (34). Nevertheless, analysis beyond the lung function tests described above requires a peripheral blood sample, likely to indicate an acute phase response. Studies from China, almost all confirmed in Europe, point to a number of laboratory abnormalities, although many are to be expected as part of the response to viral infections (Table 5). Increased levels of markers of the acute phase response (typically, C-reactive protein), and levels of cytokines (including IL-6, IL-8, IL-10 and tumour necrosis factor-α), led to the concept of the cytokine storm (also described as the cytokine release syndrome), an expression first used in graft versus host disease (35) but also used in the context of inflammatory connective tissue disease, influenza, and SARS-CoV-1 (36–39). This principle suggests the broader pathological effects of a SARS-CoV-2 infection are mediated by excessive levels of pro-inflammatory cytokines. A corollary of this theory predicts that targeting key cytokines is an effective treatment, as appears to be the case for IL-6. Zhang et al. have hypothesised that the benefit of JAK-STAT inhibitor baricitinib in patients with active rheumatoid arthritis and systemic lupus erythematosus may benefit those with a severe COVID-19 infection (40), as patients with these conditions (41) likely to be experiencing a cytokine storm.

**TABLE 5:** Laboratory abnormalities in COVID-19.

Increased levels/numbers	Total white cell count, neutrophil count, erythrocyte sedimentation rate, lactate dehydrogenase, aspartate aminotransferase, D-dimers, soluble P-selectin, creatine kinase, pro-calcitonin, IL-6, ferritin, C-reactive protein, troponin-I, N-proBNP.
Decreased levels/numbers	Lymphocyte count, CD3+ve, CD4+ve and CD19+ve lymphocytes, platelets, albumin, sodium, haemoglobin, urea.

From references 13–18 and elsewhere

### Coagulopathy

Arterial and venous thromboses are potential consequences of a COVID-19 infection (41). Increased levels of D-dimers, alongside thrombocytopenia, increased soluble P selectin (reflecting platelet activation), with prolonged prothrombin and partial thromboplastin times, may be interpreted as being linked to this increased risk (41–43). A small study reported the incidence of thrombotic complications in those critically ill patients to be 31% (44). The precise aetiology of the coagulopathy is unclear, but may be in part due to the cytokine storm and/or altered vascular function.

A healthy endothelium is essentially anti-coagulant: a damaged endothelium is pro-coagulant. The likelihood of endothelial cell damage is suggested by increased levels of von Willebrand factor and soluble thrombomodulin (45), although the origin of the increases (lung and/or general circulation) cannot be determined. Nevertheless, an increased von Willebrand factor may contribute to thrombosis by cross-linking platelets (46). Given the above, there is considerable interest in prophylactic and therapeutic anti-coagulant and anti-thrombotic therapy (47). However, although a moderate dose (150 mg) of aspirin daily in patients admitted to hospital resulted in a lower incidence of thrombotic events and a higher proportion discharged alive, there was an increased incidence of haemorrhage (48).

## Epidemiology of COVID-19 in the United Kingdom

### Risk Factors Revisited

In one of the largest studies on the UK population, Clift et al. (49) reported data on over 6 million persons from 1,205 General Practices in England. Of these, 10,776 [mean age (standard deviation) 69.6 (17.9) years, 55.5% men] were admitted to hospital with a proven SARS-CoV-2 infection, and of whom 4,384 [age 80.3 (12.1) years, 57.4% men] subsequently died. Risk factors for hospital admission and death are shown in Table 6, ranked by hazard ratio and headed by Down’s syndrome, renal transplantation, both types of diabetes and residential or care home. Interestingly, with an adjusted hazard ratio of 0.84 (95% CI 0.73–0.97), asthma brings a significant reduction in the risk of death in women, but not in men [HR (95% CI) 1.03 (0.91–1.17)], whereas it contributes to an increased likelihood of admission in both sexes.

**TABLE 6:** Risk factors for hospital admission and death in England in a COVID-19 infection.

Risk factor	Admission (men)	Admission (women)	Death (men)	Death (women)
Down’s syndrome	4.36	8.84	9.80	32.55
Renal transplant	7.09	5.54	3.20	7.84
Type 1 diabetes	3.66	4.03	5.84	4.02
Type 2 diabetes	2.57	2.64	4.74	6.29
Residential or care home	2.52	1.84	4.28	3.61
Chronic kidney disease	5.03	2.75	2.27	2.09
Cerebral palsy	2.85	2.66	2.77	3.45
Dementia	2.12	1.73	3.14	2.91
BAME	2.08	1.79	2.16	1.56
Epilepsy	1.75	1.57	1.60	1.58
Social deprivation score	1.46	1.52	1.50	1.48
Congestive cardiac failure	1.33	1.38	1.40	1.37
Chronic obstructive pulmonary disease	1.36	1.34	1.25	1.50
Stroke	1.31	1.39	1.24	1.34
Peripheral vascular disease	1.27	1.21	1.38	1.42
Severe mental illness	1.28	1.37	1.26	1.29
Thromboembolism	1.30	1.34	1.36	1.18
Atrial fibrillation	1.19	1.34	1.11	1.18
Coronary heart disease	1.06	1.11	1.13	1.24
Asthma	1.10	1.12	*	0.84
Blood cancer	1.29	1.40	*	1.50
Respiratory system cancer	1.44	1.65	*	1.70
Parkinson’s disease	2.05	1.70	1.93	*
Rheumatoid arthritis or SLE	1.30	1.35	*	1.32
Liver cirrhosis	1.88	1.83	*	1.85

Data are adjusted hazard ratios for the particular risk factor. All are significant at *p* < 0.05 except*. BAME, black, Asian, and Minority Ethnic; SLE, systemic lupus erythematosus. Modified from Clift et al. (41).

These data, in addition to age, sex, UK postcode (as a marker of social deprivation), rare lung conditions (such as cystic fibrosis), body mass index and other features were used to model a calculator for the risk of COVID-19 associated hospital admission, and death (50). With this model, a 67-year-old white British man with coronary artery disease, a BMI of 25.8, and no other risk factors has an associated risk of hospital admission of 0.1394% (1 in 717) and an 0.0444% risk of death (1 in 2252). Compared to an age-matched man free of these risk factors, these bring relative risks of 1.045 for admission and 1.13 for death. Similarly, a 57-year-old Indian woman with a non-renal solid organ transplant on immunosuppression (requiring shielding in 2020) and a BMI of 25.0 with no other risk factors has risks of 0.405% (1 in 247) for admission and 0.039% (1 in 2564) for death. Compared to an ethnicity and age-matched woman free of these risk factors, these bring relative risks of 8.22 for admission and 6.96 for death. These data are likely to be modified in those who are vaccinated, in that those who are vaccinated are less likely to be infected, admitted to hospital, or die.

### The Three Waves

A view of the WHO’s COVID-19 dashboard (2) shows four cycles of cases, with peaks in January, April, and August 2020 and January 2022 (and possibly in March 2022) (Figure 1), the precise reasons for which are unclear but may reflect the differing infectivity of variants of the original SARS-CoV-2. Similarly, there were peaks in the number of deaths in January (103,228 in the week of 18 January), April (96,071 in the week of 26 April), and August 2021 (71,012 in the week of 23 August), with a fourth in February 2022 (76,118 in the week of 7 February), which may reflect a combination of the infectivity and lethality of the variants.

**FIGURE 2:**
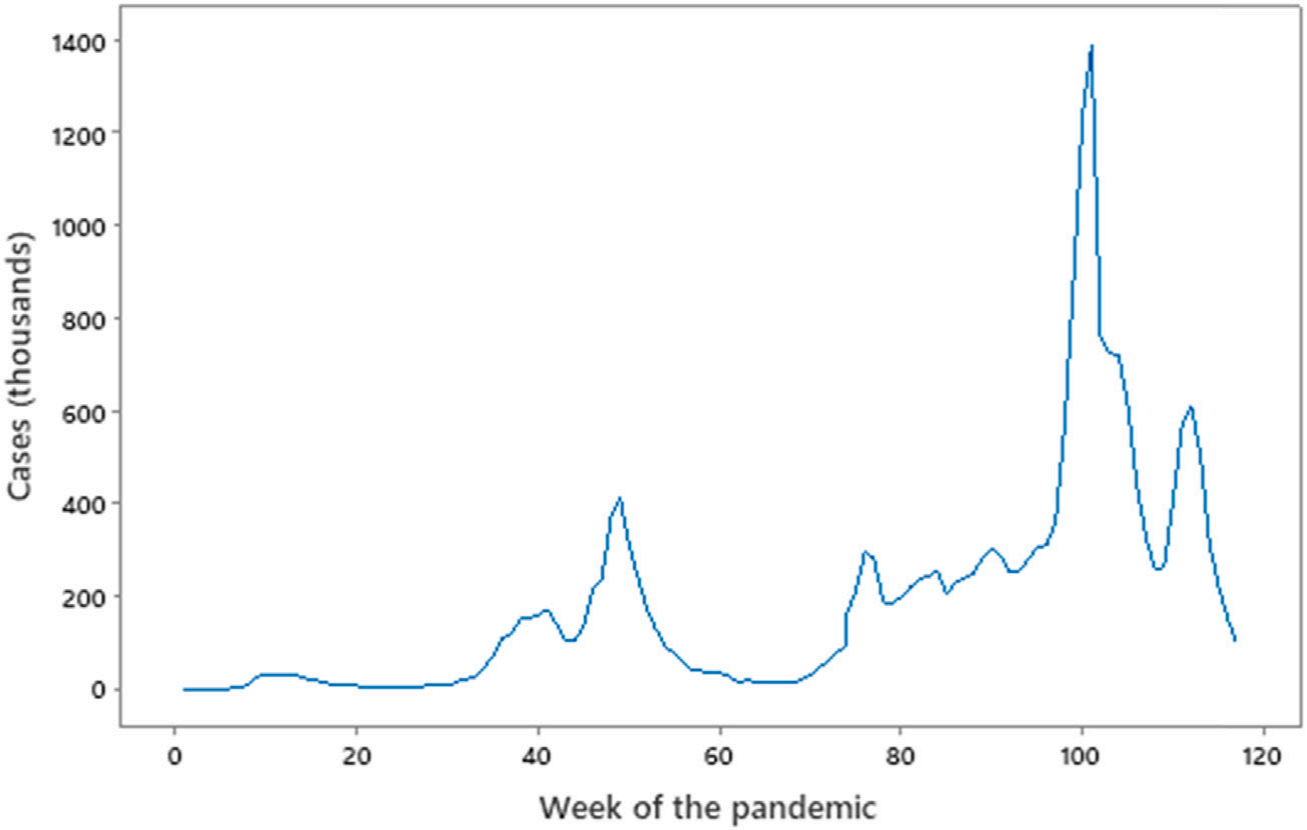
Cases vs. Week of the pandemic. Source: Office for National Statistics (http://ons.gov.uk). Week 0 = 3 February 2020, Week 60 = 22 March 2021, Week 120 = 16 May 2022. Note the failure of the peaks to overlap in sequence with data in Figure 3, and the very minor peak of the first wave (week 10) compared to the peaks that follow. The three sub-waves from week 75 are clear.

**FIGURE 3:**
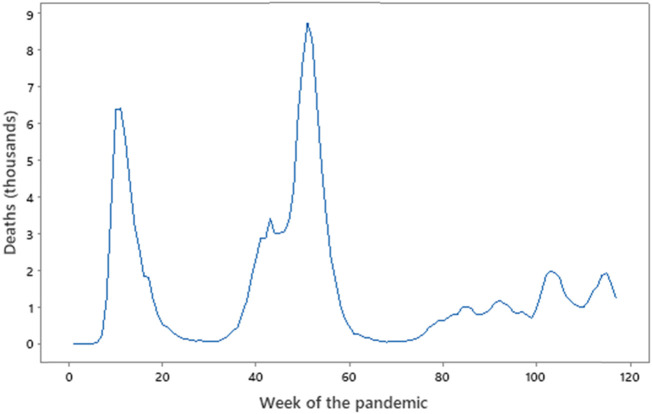
UK deaths vs. Week of the pandemic. Source: Office for National Statistics (http://ons.gov.uk). Week 0 = 3 February 2020, Week 60 = 22 March 2021, Week 120 = 16 May 2022. Note the failure of the peaks to overlap in sequence with data in Figure 2. Deaths in the third wave from week 80 are small compared to waves one and two.

The UK also experienced cycles, described as waves, some at different times to those globally. The key metrics provided by the WHO are the number of confirmed UK cases (Figure 2) and number of deaths (Figure 3), and although these do not align perfectly with global waves. Nevertheless, taking a cut-off point as the trough in deaths between the peak, the pandemic in the UK may be considered as having lasted from February to August 2020, September 2020 to mid-May 2021, and mid-May 2021 to the spring of 2022. Each had its own character—the first, lasting perhaps 31 weeks, was dogged by inexperience with this form of an acute pandemic and a lack of general overall planning and best-practice responses (the first rapid NICE guidelines being published in April 2020). The second wave of perhaps 37 weeks saw two national lockdowns, the first being most of November 2020 and the second from early January to early March 2021.

The third wave was prolonged and, unlike its predecessors, had three sub-waves. Starting in June 2020, the first sub-wave’s infections, admission and deaths were reasonably constant up to the end of November in which month there were 241,000 cases/week and 985 deaths/week: a case fatality rate (CFR) of 0.4%. The second, lasting from December to February, saw a great increase in the number of cases at 655,406/week, but with a smaller increase of 1,311 deaths/week, and so reduced CFR of 0.2%. The end of February saw the change from the second to third sub-wave of March and April, which saw 356,377 cases/week and 1485 deaths/week with a CFR of 0.4%.

**FIGURE 4:**
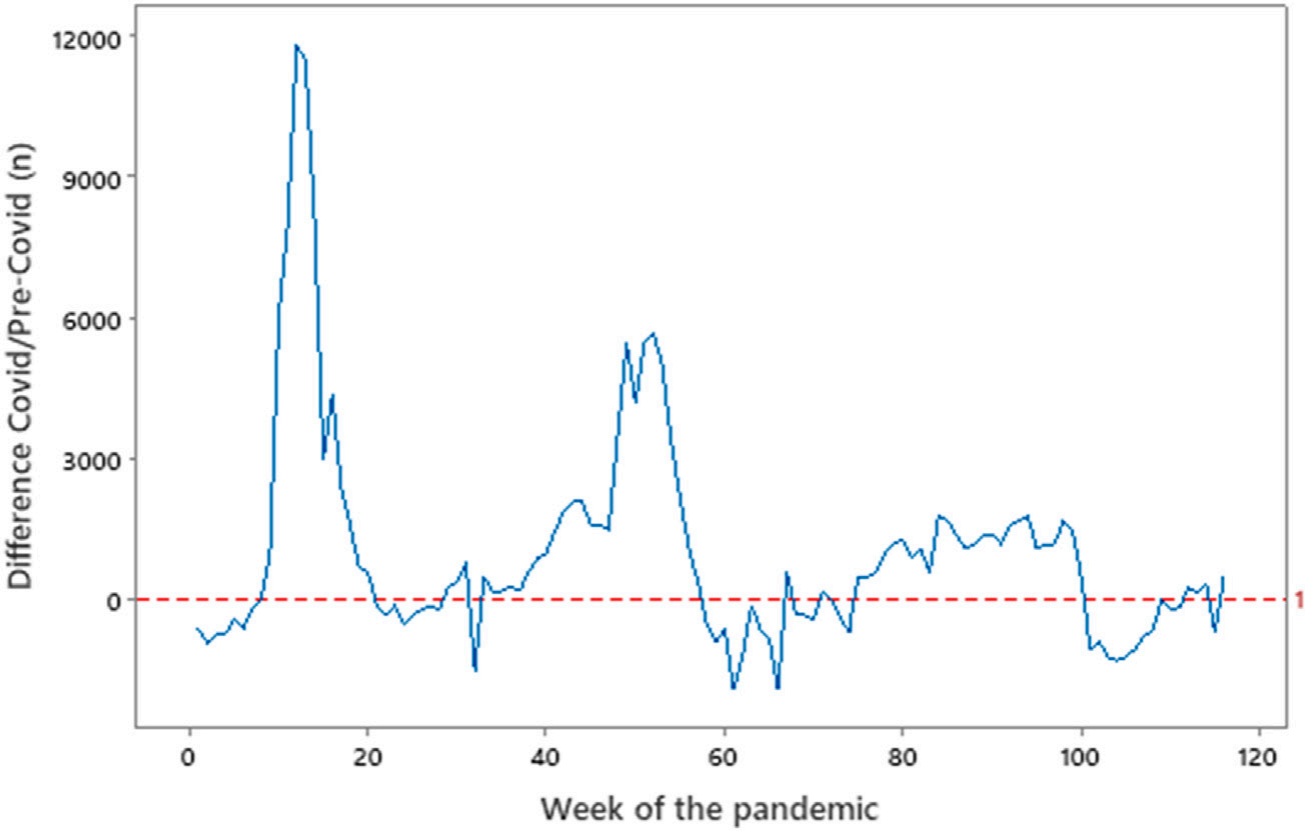
Deaths in England and Wales in the Covid vs. Pre-Covid periods. Source: Office for National Statistics (http://ons.gov.uk), data from England and Wales. Week 0 = 3 February 2020, Week 60 = 22 March 2021, Week 120 = 16 May 2022. Where the line dips below zero, there were fewer Covid deaths in 2020–2021 than in 2015–2019.

Table 7 shows the number of cases and deaths in each wave in comparison to the average rates of death for the equivalent pre-Covid period during 2015–2019. A number of observations are pertinent. In the first wave, the number of deaths exceeds the number of cases probably because of failure to correctly describe a case, itself due to lack of awareness and objective scientific testing. Although imperfect, deaths in England and Wales due to COVID-19 may be represented by the difference in the number of deaths compared to those for the same period in the pre-COVID-19 era (2015–2019) (Figure 4). These data point to over 54,000 excess deaths in the first wave, over 43,000 in the second, and over 21,000 in the third (the latter up to April 2022). However, in the first 12 weeks of 2022, there were 141,000 deaths compared to 175,000 in 2021 and an average of 149,000 in the 5-year pre-COVID-19 period.

**TABLE 7:** Cases and Deaths in the three COVID-19 waves in the UK

Dates^a^	Wave^a^				
	1st	2nd	3rd part 1	3rd part 2	3rd part 3
	February–August 2020	September 2020–Mid-May 2021	Mid-May 2021–November 2021	December 2021–February 2022	February 2022–April 2022^d^
Duration (weeks)	31	37	28	17	9
Total UK Cases (n, millions)^b^	0.34	4.11	5.93	8.26	3.15
Mean UK Cases/week (thousands)^b^	11.0	111.1	211.9	688.4	0.35
Deaths 2015–2019^c^ (thousands)	310.7	393.8	265.0	145.5	84.9
Deaths 2020–2021^c^ (thousands)	365.5	436.8	289.6	142.4	85.2
Difference in deaths/week (n)	+1,767	+1,162	+879	−259	+40
Difference in deaths/week (%)	+17.6	+10.9	+9.3	−2.1	+0.4

a Other sources give alternative dates (e.g., refs 156–158).

b UK government figures.

c ONS data from England and Wales.

d Cut-off on 30 April 2022.

Time will tell if there will be a fourth wave. The infection is, as with influenza, destined to become endemic and calls for annual vaccinations (51–54). This will be tempered by the grim knowledge of a potential additional variant resistant to these vaccinations.

## Management of COVID-19

In many respects, it could be argued that SARS-CoV-2 is “simply” another virus, especially from the structural point of view, and as a pathogen it shares much with many other viruses—influenza comes to mind. For example, as with others, there is a spectrum of its effects on a population, from asymptomatic, *via* pneumonia, to lethal (although not as lethal as some), and its detection and management have much, if not all, in common with other respiratory viruses. Some of this variability may be explained by risk factors, particularly diabetes, hypertension, and COPD, but there is growing evidence on immune response genes, particularly in HLA and cytokine responses, whilst genome-wide association studies have identified loci at 3p21.31 and 9q34.2 that are linked to severity (55). Nonetheless, treatment follows symptoms, developing from general medications for mild disease to mechanical respiratory support for those with hypoxic acute respiratory distress syndromes.

Proven pharmaceutical treatments for those in hospital include immunosuppression with dexamethasone and tocilizumab (targeting the IL-6 receptor), the anti-viral agent molnupiravir (acting on the viral RNA polymerase), and the selective serotonin reuptake inhibitor fluvoxamine (56–59). The WHO and the UK’s National Institute for Health and Care Excellence (NICE) have published guidelines (60, 61). Ritonavir, a protease inhibitor used to treat HIV, is effective in reducing admissions to hospital in those in the community with mild to moderately-severe disease (62). Other treatments include monoclonal antibodies targeting the viral spike protein, which must be infused intravenously (63, 64). One such product, sotrovimab, trialled in non-hospitalised high-risk patients with mild to moderate disease, was linked to a reduction in the primary outcome of all hospitalisations (adjusted relative risk 0.21, 95% CI 0.09–0.50) and four out of five secondary outcomes (including reduced emergency department visit, reduction in viral load, and progression to severe disease) (65). A similar study in patients with mild to moderate disease found that treatment with bamlanivimab and etesevimab was linked to a significant reduction in hospitalisations and visits to an emergency department alongside a reduction in viral load (66).

From December 2021, molnupiravir and the monoclonal antibody Ronapreve were made available to individuals who were positive for SARS-CoV-2 and who were at the highest risk of COVID-19, many of which are shown in Table 6. These include people with Down’s syndrome, multiple sclerosis, motor neurone disease, Huntington’s disease, myasthenia gravis, sickle cell disease, certain types of cancer, HIV or AIDS, a severe liver condition such as cirrhosis, a recent organ, bone marrow or stem cell transplant, a condition likely to lead to infections, and certain types of chemotherapy and radiotherapy within the last 2 weeks (67).

## Vaccination

### The Vaccines

By early April 2022, the WHO reported 276 vaccines in development, 109 in clinical testing, and 24 in use (68), although four dominate in the United States and Europe. Two of these are based on viral vectors (those from Oxford/Astra Zeneca and Janssen/Johnson & Johnson), and two deliver mRNA within a lipid nanoparticle (produced by Pfizer/BioNtech and by Moderna) (1). Those widely used in the UK are those of Oxford Astra-Zeneca (ChAdOx1), Moderna (mRNA-1273), and Pfizer-BioNtech (BNT162b2) (69–71), with several systematic reviews available on these and other vaccines (72–74).

Although differences in the format of clinical trials (age, sex, co-morbidities, etc.) bring difficulties in direct comparison between the vaccines, an independent field study reported 28–34-day vaccine effects against hospital admission of 88% (95% CI 75–94) and 91% (75–84) for the Oxford Astra-Zeneca and Pfizer-BioNtech vaccines, respectively (85). Of 2,572,008 Scottish people with at least one vaccination, 1,196 (0.046%) had severe COVID-19, 883 (0.034%) were admitted, and 541 (0.021%) died. Of those receiving the Oxford Astra-Zeneca and Pfizer-BioNtech vaccines, severe COVID-19 outcomes were found in 0.41% and 0.53% respectively. However, in those aged 80 years and older, the Pfizer/BioNTech vaccine brought an event rate of 6.3%, compared to 1.06% in those vaccinated with the Oxford/Astea Zeneca product (86). Either product was used in mass-vaccination of the UK population in 2021 using two doses, although heterologous vaccination (two vaccines of different biology) also provides a robust immune response (87). By 10 April 2020, 91.9% of the eligible UK population had received their first dose, 86.1% their second dose, and 67.7% a third dose (88). Around this time, a major university teaching hospital in the Midlands reported that 90% of its staff had received one dose of a vaccine, 86% two, and 57% three doses, whilst 69% of their patients had been vaccinated. The Moderna mRNA product (71) entered the UK vaccination programme during the summer of 2021.

### Side-Effects

Despite the clinical effectiveness of the vaccines, as with any drug, and certainly with other vaccines, there were notable adverse reactions, which vary in severity between the different vaccines (Table 8). In almost all instances, the frequency of reported side effects was inversely proportional to age (89, 90). The Pfizer/BioNtech product causes around 11 allergic reactions per million doses so it should not be used in those with hypersensitivity issues. Similarly, the Moderna mRNA vaccine links to 2.5 cases of anaphylaxis per million doses (91, 92). Those vaccinated with Oxford/Astra Zeneca and Janssen/Johnson & Johnson products are at risk of venous thrombosis and/or thrombocytopenia (subsequently named vaccine-induced immune thrombotic thrombocytopenia, VITT) at frequencies of 7–10/million and 3/million, respectively (93). Although there is no exact parallel in non-viral pathology, the nearest conditions, immune thrombocytopenia purpura and thrombotic cytopenic purpura, present at frequencies of around 27/million and 11/million respectively. The precise pathophysiological link between the vaccine and VITT is unclear, but the thrombocytopenia can be accounted for by antibodies to platelet factor 4, as are present in heparin-induced thrombocytopenia. Klok et al. (94) describe VITT as an autoimmune condition, with anti-PF4 antibodies contributing to platelet activation and platelet microparticle release, and so a consumptive coagulopathy that accounts for the thrombocytopenia, but also possible hypofibrinogenaemia. NICE has published a guideline (NG200) on the diagnosis (based specifically on a platelet count, which, if low numbers are found, should be followed up with a coagulation screen) and management of this condition (such as the possible need for anticoagulation, probably with a non-heparin drug) (95).

**TABLE 8:** Frequency of side effects in three vaccines.

	Pfizer/BioNTech		Moderna		Johnson & Johnson	
	Mild to moderate	Severe	Mild to moderate	Severe	Mild to moderate	Severe
Injection site pain	71.1	0.85	86.7	3.6	30.9	0.3
Headache	43.4	1.85	54.6	4.0	37.4	0.65
Fatigue	51.2	3.7	63.0	8.7	36.7	1.0
Myalgia	32.5	1.6	54.1	7.8	31.5	0.8
Vomiting	1.1	0.15	—	—	—	—
Nausea	—	—	16.5	0.2	13.9	0.15
Fever	25.8	0.75	13.8	1.0	7.9	0.2

Data are % of patients reporting a side effect.

A perspective of COVID-19 vaccines against other vaccines and the dangers of not vaccinating is warranted. A case series of reports estimated additional myocarditis cases at 1 to 2 per million for the first dose of the AZ/ChadOx1 and Pfizer/BNT162b2 vaccines and 6 per million for the Moderna/mRNA-1273 vaccine. These risks should be considered against the risk of 40 myocarditis events per million during the month after a SARS-CoV-2 infection. Similarly, the pre-pandemic incidence of clinical myocarditis in the general population is around 13/million. The incidence in those hospitalised and unvaccinated with SARS-CoV-2 is 2261/million, but the incidence in those vaccinated = 21/million (76). The incidence ratio for pulmonary embolism has been reported as being 1.21 (95% CI, 1.07–1.36) after vaccination with BNT162b2, consistent with that seen after ChAdOx1 vaccination. Notably, the increased risk of this possibly fatal thrombosis in COVID-19 patients is 15.31 (95% CI 14.08–16.65) (77).

In an analysis of 48,159 individuals, 22.6% vaccinated with an anti-SARS-CoV-2 product reported an adverse event. This figure is exceeded by the 25.7% reporting an adverse event after influenza vaccination and 25.1% after a Herpes zoster vaccination. Of 268,438,666 doses of a SARS-CoV-2 vaccine administered, 227,616 adverse events were reported—a rate of 0.08%. This data included 4110 deaths, a frequency of 0.0015%, which compares with the case fatality rate defined by the WHO (at the time) in those infected with SARS-CoV-2 of around 1.8% (78). However, of more than 8 billion doses of COVID-19 vaccines administered worldwide, Maiese and others found only 55 cases of death temporally correlated with vaccination, and in 17 of these a causal relationship has been excluded (79).

### Boosting Vaccination

Developing data indicated that certain high-risk groups (estimated at 1.3 million people), such as those immunosuppressed (80), failed to generate a sufficiently high titre of protective antibodies, these being linked to vaccine efficiency (81), and were therefore at risk of infection. One study pointed to falling titres of antibodies with age: at day 119 after vaccination, a median titre of 235,228 IU/ml in those aged 18–55, 151,761 IU/ml in those aged 56–70 and 157,946 IU/ml in the over 70s (82). As isolated studies in selected groups and case reports indicated that a third boosting vaccination in these groups was effective in increasing immune responses (83, 84), this strategy was rolled out in the UK in the Autumn of 2021. The protocol was as effective as in the original vaccinations, i.e., those at highest risk first, followed by other groups. By 10 April 2022, over two-thirds of the eligible UK population had received a booster dose or third dose (88), a process enhanced by the appearance of the Omicron variant in December 2021 (see below). In the spring of 2022, the most vulnerable (e.g., those immunosuppressed) were offered a fourth dose, some with the Moderna vaccine.

### Effects of Vaccination

A study from Israel indicated that, in all age groups, as vaccine coverage increased, the incidence of SARS-CoV-2 outcomes declined (96), whilst as early as May 2021, the National Institutes of Health estimated that vaccination had prevented 140,000 deaths in the United States (97). In September 2021, the UK Health Security Agency estimated that between 119,500 and 126,800 deaths, over 230,000 hospitalisations, and around 24 million infections had been prevented by vaccination (98, 99). Of 1.24 million users of a COVID symptom reporting app who had a single vaccine dose, 0.5% subsequently tested positive for SARS-CoV-2, a figure falling to 0.2% from 971,504 who had two doses (98). Furthermore, almost all symptoms were reported less frequently in infected vaccinated individuals than in infected unvaccinated individuals, and vaccinated participants were more likely to be completely asymptomatic, especially if they were 60 years or older (100).

An unanswered question is the longevity of the effects of the vaccines, which by definition is (as yet) unknown. Regular testing for antibody and cellular responses will determine any such effects and thus also the need for re-vaccination (101, 102).

## Variants

Soon after the genome of SARS-CoV-2 was published, often described as the Wuhan, wild-type, or ancestral genome, local variants were reported, reflecting the natural mutation rate in any simple organism. The most important mutations are those leading to changes in the tertiary structure of the spike protein, a highly-glycosylated 1273 amino acid, 180–200 kDa trimer by which the virion binds ACE2 (4–9). The protein binds to target cell ACE2 *via* residues 319–541 on the S1 subunit, which make up the receptor binding domain, the entire subunit being amino acids 14–685 (103).

One of the first variants of potential importance was a single nucleotide polymorphism (SNP) causing the change of aspartic acid to glycine at position 614, hence D614G, described in April 2020 (104). This mutation, although outside the receptor-binding domain, increased spike density and infectivity (105). As they appeared, the international consensus named variants by Greek letters of the alphabet and a code of the letter B followed by a numerical designation based on molecular genetics (the Phylogenetic Assessment of Named Global Outbreak; PANGO) (106).

In the UK, the identification and spread of variants were monitored by the COVID-19 Genomics UK consortium (107). Early variants were named B.1 and B1.1, although perhaps the first to generate international attention was B.1.177, characterised by an SNP mutation leading to A222V in the spike protein’s N-terminal domain, which appeared in Spain in the summer of 2020 (108). By November 2020, it became prevalent in 65% of all sequenced genomes in England (109). However, others rapidly appeared. Four such variants with marked clinical features appeared in late 2020 and early-mid 2021 (110) and were named “variants of concern” (VOC), whilst others of less concern were named “variants under investigation” (VUI).

### The Alpha Variant

This first VOC, also named B.1.1.7, was characterised by N501Y, in the receptor-binding domain, whilst other changes included A570D, D614G, and a deletion Δ69/70 in the N-terminal domain. Appearing in the UK in the Autumn of 2020, with an R value of 1.75 (111) and with increased transmissibility, estimated at 50%–75%, it became the dominant strain at the end of 2020, with an incidence of 98% by the spring of 2021. A further mutation was that which led to E484K, which increases viral replication in human upper airway cells and increases ACE2 affinity (112). This variant is reported to have been responsible for the peak of infections of the second wave from December to February 2021 (113), and by April 2021 the frequency of the previously dominant variant, B.1.177, had fallen to less than 10 cases a day (114).

### The Beta and Gamma Variants

B.1.351 (Beta), originally identified in South Africa in late 2020, was characterised by A701V, D215G, and D80A at various places in the spike protein and K417N, E484K, and N501Y in the receptor-binding domain (115). In early 2021, B.1.1.248 (P1, gamma) appeared in Brazil, characterised by mutations K417T, but also E484K and N501Y (116). However, developing data showed low rates of infection and low case fatalities in the UK (117), and despite which, these variants remained VOCs through to December 2021 (118)

### The Delta Variant

Variant B.1.617 was first described in India in late 2020, appeared in the UK in March 2021, and was classified as a VOC in April 2021 with at least three sub-variants, the most important being B.1.617.2, the variant B.1.617.1 being named Kappa. The defining SNPs for Delta produced T19R, L452R, T478K, P681R, and D950N in the spike protein; S26L, M18T, V82A, and T120I in an open reading frame; and D63G, R203M, and D377Y in the nucleocapsid (119). Notably, this variant did not contain the N501Y or E484K mutations of previous variants, enabling easier laboratory detection.

With an increased transmissibility of 60% and an R-value estimated as 7 (120), this variant displaced B.1.1.7 to become the dominant strain in the UK in July 2021. Although the case fatality rate was only around 10% of that of B.1.1.7 (121), it was reputed to be responsible for the early phase of the third wave in the late summer of 2021 (114). However, this data focussing on death (Table 7) fails to address the issue of out-of-hospital morbidity, although the Delta variant brought an increased adjusted HR (95% CI) for hospital admission versus the Alpha variant of 2.26 (1.32–3.89), with a similar trend for emergency care attendance or hospital admission [1.45 (1.08–1.95)] (122). A further notable statistic of this analysis from England only is that the white/Black/Asian ethnicity make-up of those positive for Alpha was 74.8%, 2.5%, and 14.8%, respectively, whilst of those positive for Delta, the proportions were 48.5%, 3.8%, and 37.8%, respectively, a difference of exceptionally high statistical significance (chi-squared = 2667.1, *p* < 0.00001) (122). However, this potentially biased raw data is unadjusted for other factors known to influence outcomes, such as age, sex, vaccination status, and index of multiple deprivations, so an adjusted significance is likely to be different.

### The Omicron Variant

VB.1.1.529 emerged in Botswana, Hong Kong, and South Africa in November 2021 and was named a VOI on 26 November (123). It was of interest as it carries 32 spike protein mutations (mostly SNPs, some deletions), several of which were within the receptor-binding motif formed from amino acids 333–537, i.e., S371L, S373P, S375F, K417N, N440K, G446S, S477N, T478K, E484A, Q493R, G496S, N501Y, and Y505H (124, 125). It was argued that these mutations may change the behaviour of the variant as regards transmissibility, infectivity, immune escape, and pathogenicity, as was the case of other variants, and Omicron was designated VOC a week later (126, 127). The variant was estimated to have a reproduction number of around 10 (120), that being over three times that of the Delta variant (128), amply explaining its rapid dissemination.

On 12th December 2020, 6 people in England were infected with the Beta variant, rising to 95 people a month later. Similar data for the Gamma variant was 5 and 12, and for the Delta variant, it was 6 and 156 cases. By contrast, on 12 November 2021, there were 5 cases of an Omicron infection, but an astonishing 15,287 a month later (129). On 30 December, very early data on 198,348 cases in England reported 815 hospitalisations (0.41%) and 54 deaths (0.027% of cases, 6.6% of admissions) (130). However, these data fail to provide information on those taken out of the workforce by an infection, a topic particularly relevant for front-line hospital staff who were most at risk of infection and whose absence placed a strain on the care of those admitted with any health issue.

At the global level, the Omicron variant is a likely cause of the considerable increase in the number of cases, with new records being set. The previous weekly record was 5.7 million cases in April 2021, surpassed by the 10 million confirmed cases on 27 December 2021, 15.8 million on 3 January 2022, 20.5 million on 10 January, 23 million on 17 January, and 23.3 on 24 January, levels falling thereafter but with a small increase in mid-March 2022 of 12.7 million weekly cases (2) However, this increase in cases was not linked to a parallel increase in the number of deaths, which peaked at around 76,000 in early February 2022. Thus, the case fatality rate fell from around 2.7% at the end of January 2021 to 0.3% a year later (2).

Taking the viral-neutralising titre of serum towards an early pandemic strain of the virus to be 100%, the median neutralising titre towards the Beta, Delta and Omicron variants was 31.6%, 39.1% and 7.5%, respectively in subjects double-dosed with the Oxford/Astra-Zeneca vaccine. Similarly, titres in those double-dosed with the Pfizer/BioNtech vaccine were 34.7%, 84.4%, and 3.4%, respectively. These data imply the possibility of reduced effectiveness of these vaccines against the Omicron variant, which may part-explain its highly contagious nature (131). Despite this very high rate of infectivity, Omicron infection carried an adjusted HR (95% CI) for presentation to emergency care or hospital admission of 0.62 (0.55–0.69) compared to the Delta variant, and 0.38 (0.30–0.50) for hospital admission alone (129).

### The Omicron BA.2 Variant

This variant of a variant was first described as a global (but not the UK) variant upon monitoring on 10 December 2021, but a week later it had moved to a UK variant upon monitoring and a VUC on 23 December (129, 132, 133). It was designated a VIU on 19 January 2022 and a week later was noted to have an increased growth rate, described at ‘substantial’, compared to the original Omicron BA.1 variant, with a doubling time (100%) of around a week although this settled a little to 80% (134). Subsequent data from 25 March 2022 showed a hazard ratio of 0.91 (95% CI 0.85–0.98) for risk of hospitalisation due to BA.2 to be slightly reduced compared to BA.1 (135).

An early population study of 2,623 cases of BA.2 infection reported no differences in hospitalisation of 423 people due to BA.2 compared to BA.1 with an adjusted risk ratio (95% CI) of 1.2 (0.93–1.54) or in vaccination history (136). However, a small study of 207 young (median age 39) patients positive for BA.2 and 2,793 age- and sex-matched patients positive for BA.1 found hospitalisation rates of 6.3% and 1.45, respectively (*p* < 0.0001) (137).

Molecular genetics of these viruses found new mutations leading to T376A, D405N, and R408S in the receptor-binding domain of BA.2 (136). These differences seem likely to be responsible for the differences in binding activities of monoclonal antibodies towards BA.1 and BA.2, as has been shown for the Delta variant (138). However, others found equal median titres of neutralising antibodies to both variants in those who received a BNT162b2 booster vaccine (139), whilst another group reported that currently available vaccines might be more effective against BA.2 and BA.1 (140).

### Variants Under Investigation

As is implicit, these variants are (when first recognised) merely of interest, and are under surveillance, perhaps because they have limited clinical relevance. Nevertheless, some may be responsible for admissions and even some deaths. By February 2020 five VIUs were reported, named Zeta, Eta, Theta, Kappa and Lambda, with others (Epsilon and Iota) being monitored (116). Of these, Eta and Kappa were the most clinically relevant, with 443 and 446 cases, and 12 and 1 death, respectively. By November 2021 a variant of Delta (PANGO 1.617.1) and a Mu variant were listed, whilst the Zeta and Theta variants were de-escalated (123). However, at this point a new variant, VUI-21Nov-01 (B.1.1.529), was first described, soon to be a VOC as Omicron. By December 2021 the list of VIUs was reduced to three, with nine being monitored (1286). Given the experience of the past 2 years, it may be predicted that new VUIs and VOCs will emerge.

### Implications of Variants

The first wave of vaccines was based on the original Wuhan strain of the virus, leading to fears of their lack of effect on variants with different spike proteins (141, 142). Evidence in support of this hypothesis included differing sensitivities of neutralising antibodies and vaccine effectiveness against the different variants (131, 136, 143, 144). A corollary of the changing nature of the virus over time within a trial population is that it is even more difficult to compare outcomes. For example, it has been suggested that the Alpha variant was not circulating in the trials of BNT162b2, mRNA-1273, and Ad26.COV2-S, but was present in the trials of AZD1222, and of course that the Delta or Omicron variants were not present in any population in any trial (145). The same principle of spike protein variation implies certain neutralising monoclonal antibodies raised against certain variants may no longer be effective treatments for newer variants (138–140, 146).

Despite these concerns, as of the end of December 2021, the hospital admission rates and case fatality rates for COVID-19 in the UK remained low, these being around 1% and 0.1% of cases respectively (88). Furthermore, high levels of protection (>90%) from hospitalisation and mortality due to the Alpha and Delta variants are afforded by each of the three major vaccines (98). Despite the seemingly poor response of convalescent and vaccine-induced antibodies to the Omicron variant, the second part of the adaptive response, SARS-CoV-2 specific T-cell responses, are reportedly intact (147), and may help explain the relatively low pathology of the virus.

### Population Effects of Variants

Variant-specific UK government data on the first four VOCs and some VIUs are presented in Table 9 (149). Despite the high infectivity of the Delta variant, its fatality is a little over a quarter of that of the Alpha variant, whilst Omicron, is the least fatal of all variants, except one. Omicron is significantly less likely to be the cause of hospital admission than Delta (129), but the high infectivity rate with mild to moderate symptoms caused many infected persons to isolate themselves, with repercussions for employment and staffing.

**TABLE 9:** Cases and Deaths for major variants in England.

Variants	Total case number	Deaths	Deaths/Cases (%)
Alpha	226,844	4,323	1.91
Beta	990	12	1.21
Gamma	279	0	0
Delta	977,674	5,066	0.52
Eta	462	12	2.60
VUI-21FEB-04^a^	315	1	0.32
VUI-21OCT-01^b^	25,116	110	0.44
Omicron	212,019	75	0.04

Top seven data dated 8 November 2021 (ref 111).

a A sub-variant of Eta (B.1.1.318) (ref 93).

b A sub-variant of Delta (AY.4.2) (ref 99). Omicron data (England) on 31 December 2021 (https://assets.publishing.service.gov.uk/government/uploads/system/uploads/attachment_data/file/1044522/20211231_OS_Daily_Omicron_Overview.pdf).

Although seemingly with a high case fatality rate, the Eta variant, and its sub-variant VUI-21FEB-04, were de-escalated by November 2021 (123). Although a VUI, the Delta sub-variant VUI-21OCT-01 had a case fatality rate only a little less than the major Delta variant, but by December 2020, the case tally had risen to 108,507 (126).

## Long COVID

The normal distribution demands that in some, symptoms persist long after the acute phase of the illness and manifest with a wide selection of complaints.

### Definitions

As in acute disease, the most common symptoms of long COVID are fatigue (58%) and dyspnea (44%), arthralgia (27%), and chest pain (22%) (Table 10), with around two-thirds reporting more than one symptom (149–151). Symptoms may be present in various physiological areas—pulmonary, haematological, renal, endocrine, cardiovascular, neuropsychiatric, gastrointestinal, hepatobiliary, and dermatologic—pointing to the need for a multi-disciplinary approach (150, 151).

**TABLE 10:** Signs and symptoms of long COVID-19.

Body system	Signs and symptoms
Cardiovascular	Palpitations^a^, tachycardia^a^
Dermatological	Skin rash, hair loss
Ear, nose, and throat	Dizziness, earache, changes in smell (anosmia/parosmia) and/or taste (dysgeusia)^a^, sore throat, tinnitus^a^
Gastrointestinal	Abdominal pain^a^, diarrhoea^a^, constipation^a^, acid reflux^a^, loss of appetite, nausea
Immunological	Intermittent fever^a^, new allergies^a^
Musculoskeletal	Arthralgia^a^, myalgia^a^, fatigue^a^, post-exertional malaise^a^
Neurological	Cognitive impairment (“brain fog,” loss of concentration or memory issues)^a^, dizziness^a^, headache^a^, sleep disturbance^a^, peripheral neuropathy^a^, memory issues^a^
Ophthalmological	Blurred vision^a^
Psychological/psychiatric	Anxiety^a^, depression, loneliness, delirium, depression^a^
Reproductive	Altered menstruation^a^
Respiratory	Breathlessness^a^, cough^a^
Thorax	Chest pain^a^ (could be pulmonary or cardiac in origin), expectoration

From refs 115–120 and elsewhere.

a Cited by ref 119. n.b. Many signs and symptoms are also present in acute disease.

NICE recognises this in two stages: when symptoms extend from 4 weeks up to 12 weeks and then stop, “Ongoing symptomatic COVID-19” is present. The full “Post-COVID-19”may be described as signs and symptoms that develop during or after an infection consistent with COVID-19 that continue for more than 12 weeks and are not explained by an alternative diagnosis. However, “Post-COVID-19” syndrome may be considered before 12 weeks while the possibility of an alternative underlying disease is also being assessed (152). A WHO consensus document focussed on 27 signs and symptoms (153), many of which are in common with the acute phase (Table 10).

One review found little difference in the frequency of many signs and symptoms between acute and long COVID-19 (e.g., fatigue 43% versus 44% and anosmia 11% versus 10%, respectively), although the frequency of others differed (e.g., dyspnea 31% versus 40%, throat pain 6% versus 12%, and palpitations 6% versus 20%, respectively) (154). One hypothesis to explain the broad nature of these signs and symptoms is microvascular damage (155), whilst a small study of 31 subjects with long COVID and 31 age- and sex-matched controls found elevated levels of interferons and IL6 in the former, who were also more likely to have been hospitalised (156). Taking a holistic approach, a score of health-related quality of life (e.g., SF-36) was considerably worse in long COVID than in the acute phase, with a larger difference in those with co-morbidity such as for overweight and obesity, diabetes, and heart failure and in those admitted to hospital (157).

### Lung Disease

As the dominant clinical features of COVID-19 are pneumonia, pneumonitis, and ARDS, a potential severe long-term consequence is the clinically-recognised interstitial lung disease (ILD) (158). Post-infection imaging often reveals pulmonary fibrosis, characterised by excessive deposition of extracellular matrix material by fibroblast secondary to the inflammation, whilst other methods point to microvascular damage and microthrombi (159, 160), leading to (at least) COPD and emphysema.

A small study from Wuhan indicated persistent radiological changes in 24% of patients 12 months after the acute infection (161). A study from Spain of 313 patients admitted to hospital with COVID-19 pneumonia reported 55% to have objective lung function <80% of that expected at 60 days post-discharge, improving to 47% at 180 days (162). Of 67 patients undergoing pulmonary function tests an average of 15 weeks after discharge, 79% had at least one abnormal parameter, and of 72 patients who had a CT scan an average of 18 weeks after discharge, 44% showed persistent ground-glass opacities (implying increased lung parenchyma density) and 215 showed fibrosis (163). The further long-term implications of these findings are as yet unclear but may include a propensity for the development of more permanent pulmonary diseases such as emphysema, pulmonary oedema, and fibrosis and may necessitate additional referrals to secondary care.

### Cardiovascular Disease

Although SARS-CoV-2 has as yet had little impact on cancer, it may have a role in cardiovascular disease. Not only is this broad group of conditions a risk factor for infection and poor outcome, but the virus may also precipitate stroke, cardiac arrest, myocarditis, and arrhythmia (principally atrial fibrillation) whilst patients are hospitalised, all of which have long-term effects (164, 165). Damage to the myocardium (as shown by increased levels of troponins and pro-NT-BNP) (Table 5) may lead to left ventricular dysfunction and heart failure (166). As raised D-dimers in COVID-19 are indicative of arterial and venous coagulopathy, infarction events may be the consequence of microthrombi and may justify prophylactic anticoagulation (167, 168).

## Perspectives

### As a Viral Disease

A broader view of the present pandemic compares it with other such global respiratory viral pandemics and epidemics, such as those of influenza in 1918–1919 and 2009, other coronavirus epidemics (SARS-Cov-1 and MERS-CoV), and Ebola. Each has its specific components (e.g., mode of entry to the target cell) and much in common (e.g., symptoms of fever and cough, and increased levels of circulating cytokines) (Table 11) (13, 169–171). Based on these data, SARS-CoV-2 has a relatively reduced mortality rate, a relatively low hospitalisation rate, a high community attack rate and reproductive number, and an average incubation time.

**TABLE 11:** Major viral epidemics and pandemics in the modern age.

	Influenza 1918–19	Ebola	SARS-CoV-1	Influenza 2009	MERS-CoV	SARS-CoV-2^a^
Untreated case fatality rate^b^ ^,^ ^c^	3.7%	56.5%	10%	0.2%	35.5%	1.2%
Hospitalisation rate^b^	Few	57.5%	>70%	37.5%	Most cases	20%
Community attack rate	33%	17.5%	35%	15%	8.5%	35%
Reproductive number (R)	2.0	1.9	2.9	1.5	<1	3.0
Median incubation time (days)	8.5	11.5	4.2	2.5	5	5
Number of deaths	50 million	13,000	774	300,000	858	6.3 million^d^

a Ancestral Wuhan strain.

b Of those infected.

c Includes some treated cases.

d On 28th June 2022.

### As a Cause of Death

Although the overall annual global case fatality rate is small (October 2020–September 2021: ∼3.7 million) (2) when compared to other causes of death (cardiovascular disease ∼17.6 million, cancer ∼10 million), it exceeds that of other specific infectious diseases (tuberculosis ∼1.2 million, HIV/AIDS ∼1 million, malaria ∼0.72 million) (172). In the UK, the virus killed around 69,700 people in 2020, making it the third-highest cause after cancer (151,000) and circulatory disease (132,600) but exceeding that of respiratory disease (62,900) (173). Notably, in 2019, there were 10,000 more deaths from all respiratory diseases (including 5,000 more from pneumonias) than in 2020, suggesting that these “missing” deaths are due to COVID-19. Data of this nature is published by the Office for National Statistics each July. Figure 4 points to the increased number of deaths in the period 2020–2021 compared to 2015–2019 in each of the three waves.

Although Table 6 points to risk factors in admission and death, a more recent data set from over 9.6 million persons compared deaths in the pre-pandemic era with 35,369 deaths in the first wave of March–May 2020 (174). In contrast to Table 6 data (42), this study found no excess deaths due to rheumatoid arthritis, chronic heart disease or venous thromboembolism, but confirmed excess deaths due to increased age, male sex, cerebrovascular disease, dementia, chronic kidney disease, overweight and obesity (174), and South Asian and Black ethnicity (175) and that asthma is not linked to death. The study also reinforces hypertension as a risk factor, but, curiously, being a current smoker brings a reduced risk of death, and cancer diagnosed in the past year also bring a reduced risk. A further example of the compounding effects of risk factors on hospital admission and death is of Black, Asian and Minority Ethnic background (BAME), and overweight or obesity: The estimated risk of COVID-19 mortality at a BMI of 40 kg/m^2^ in white ethnicities was equivalent to the risk observed at a BMI of 30.1 kg/m^2^ in Black, 27.0 kg/m^2^ in South Asian, and 32.2 kg/m^2^ in other ethnic minority groups (176). Of 50,000 deaths during March-June 2020, only 8.9% occurred in those free of any co-morbidities (177).

With the advent of improved treatments and vaccination (Table 7), it may be predicted that the number of deaths in 2022 and beyond will be lower. In England and Wales, there were an average of around 531,000 deaths in the years 2015–2019, rising by 15.6% to some 614,000 in 2020, almost certainly due to COVID-19. However, preliminary data from January 2022 point to around 586,000 deaths, up around 10% from 2015 to 2019, which does indeed point to a smaller number of COVID-19 deaths (178).

### As a Cause of Long-Term Morbidity

A weakness of this death-centred approach is that it fails to address the likelihood of the morbidity of Long-Covid, which may be substantial, such as lung and cardiac disease described above (179). The full population impact of long COVID, by definition, is unclear, and without firm data, there can only be speculative extrapolations, although firm data will eventually appear. Nevertheless, a study of 8,591 post-COVID-19 survivors found fatigue/weakness (in 28%), arthromyalgia (26%), depression (23%), anxiety (22%), memory loss (19%), concentration difficulties (18%), dyspnea (18%), and insomnia (12%) (180). This may entail additional referrals to specialists such as rheumatologists, chest physicians and neurologists.

Interestingly, in the pre-COVID-19 period of December/January of 2018/2019 and 2019/2020, the rate of hospitalisation due to influenza was of the order of 4 per 100,000 (181). In 2020/2021 and 2021/2022 this rate was close to zero, presumably due to public health measures and that those succumbing to influenza instead caught SARS-CoV-2. Unscheduled A&E and inpatient visits for paediatric asthma fell during the pandemic, possibly due to reduced viral upper respiratory tract infections, although this may be due to parental concerns of potential adverse effects (182). The effects of the pandemic, and the morbidity it brings, on the UK National Health Service, with many staff self-isolating because of an infection or a close contact with someone infected, were profound. This therefore called for the cancellation of routine appointments and procedures that increased the waiting from 4.5 million patient events before the pandemic to 6 million, with 300,000 waiting over a year. In addition, an estimated 10 million people failed to present to the NHS during the pandemic, implying further presentations. The extra burden placed on General Practitioners in England as a result of the pandemic may part-explain the reduction in several diabetes health checks, the consequences of which are unclear but are likely to be detrimental (183) (Table 12). Globally, there was a median 55.5% reduction (58% in the UK) of in-person outpatient care utilisation in visits, diagnostic/screening procedures, and treatment in emergency care, primary care and speciality care (184).

**TABLE 12:** Diabetes health checks in 2021 compared to 2019–2020.

Health check	2021(%)	2019–2020(%)
HbA1c	73.9	93.5
Blood pressure	70.0	95.4
Cholesterol	63.0	91.0
Foot health	46.8	83.9
Body mass index	60.6	88.3
Patients receiving all care checks^a^	27.0	55.2
Patients achieving targets of HbA1c < 58 mmol/mol, cholesterol <5 mmol/L, blood pressure <140/90 mm Hg	34.9	40.3

a First 9 months of 2021. Data refers to GPs in England.

## Conclusion and the Future

The SARS-CoV-2 pandemic has forced an unprecedented global scientific and medical collaboration. As the virus has a relatively high dynamic mutation rate compared to other viruses (185, 186), new variants will inevitably appear, demanding continued international cooperation from scientists and clinicians. This is pertinent as COVID-19 is destined to become endemic (51–54, 187, 189). The second wave was characterised by a peak case fatality rate of around 2.1%, falling in the early part of the third wave to around 0.35%. The middle period of the third wave was characterised by a huge case increase due to the Omicron, a case fatality rate of 0.15%, and a fall in both metrics. The latter reductions possibly reflect the effects of vaccination and the reduced pathogenicity of the Omicron variant (Tables 7, 9). The reductions due to Omicron in the latter part of the third wave may be due to its lower replication competence in a culture of lung cells compared to the other variants (190). However, the advent of the BA.2 variant of Omicron in March 2022 saw a resurgence in cases and deaths, with the case fatality rate rising to 0.25% (2).

The early identification of those at risk of possibly fatal disease progression remains an important goal, with laboratory markers (e.g., D-dimers and lymphopenia) and imaging (e.g., ground-glass opacity) being important. As regards the former, miRNAs may be an additional useful tool (191, 192).

This reduction in hospital admission and deaths in January 2022 lead to an easing of the public health initiatives in February 2022, and the removal of all restrictions at the end of that month, although some insist on the wearing of face masks (156). Indeed, despite a very high case rate, the pre-COVID-19 year of 2015–2019 saw a mean of around 52,000 deaths in January: the figure for January 2022 is around 50,800 (although possibly due to mild winter weather). However, the remaining issues are the likelihood of the infection being an established health issue, and the unknown effects of long Covid. Globally, whilst both cases and death continue to rise, the case fatality rate continues to fall (Table 1) (2). A further feature is the realisation of the difference between deaths involving a particular pathology, and that the death is due to that pathology. For example, in January 2022, there were 16,924 deaths involving the respiratory system, but only 5606 of these (33.1%) were due to respiratory disease. Similarly, there were 9151 deaths involving influenza and pneumonia, but 1746 (19.1%) were due to these pathologies. In contrast, of the 5,173 deaths involving COVID-19, a much higher proportion (3850, 74.4%) were due to this infection (148). As more and more of us are infected with SARS-CoV-2 (in early February 2020, at least 26.2% of the UK were infected, and this percentage is very likely to rise), this will become increasingly pertinent (193).

The appearance of SARS-CoV-2 and the infection (COVID-19) it causes has unified international medico-scientific communities and brought an unmatched awareness of health issues into all corners of human society. The early prediction that the pandemic would last until the spring of 2021 proved incorrect, with 7.9 million UK hospitalisations and 260,000 deaths (193, 194). Nevertheless, the full extent to which it will have a major and long-lasting negative effect on the individual and society in the UK and globally remains to be seen.

### Notes Added in Press

Since resubmission of this review on 12th May 2022, several new and important features of the infection have arisen. As of 28th June, globally, the weekly number of cases remained around 3.5 million, but deaths have continued to fall, with the case fatality rate also falling to 0.19% (2). In January 2021, this latter figure was 2.4%. In the UK, these data were around 92,000 cases/week, 250 deaths/week, and so a case fatality rate of 0.27%, compared to around 2.0 in January 2021 (2).

According to the Office for National Statistics, in England and Wales, in the last 2 weeks of May and first 3 weeks of June, the number of deaths due to influenza or pneumonia (*n* = 1689) was significantly higher than the deaths due to COVID-19 (*n* = 1036, *p* = 0.028) (177). The number of all-cause deaths in the first 24 weeks of 2022 (up to 17th June, mean 11,169 standard deviation 1,285) was not statistically different from those in the same 24 weeks of the pre-COVID-19 era of 2015–2019 (11,106, 1,636, *p* = 0.783). This equates to 1,526 more deaths in 2022 than the average for 2015–2019. Since the same spreadsheets report 20,042 deaths involving COVID-19 over this time period, it follows that there must have been a considerable reduction in other deaths to account for this difference. Nevertheless, these data added to the hypothesis of the end of the Omicron BA.1/BA.2 sub-pandemic (196).

Despite the above, there was a modest increase in cases, deaths and hospital admissions in the middle of June 2022 (88). This was reputed to be linked to Omicron variants BA.4 and BA.5 (comprising 22.3% and 39.5% respectively of new cases), and variant XE, a recombinant of BA.1 and BA.2) (197). In the USA, variant BA.2.21.1 became the dominant species as of 25th May 2022 (195), but had yet to make an impact in the UK.

## References

[1] Salvamani S Tan HZ Thang WJ Ter HC Wan MS Gunasekaran B Understanding the Dynamics of COVID-19; Implications for Therapeutic Intervention, Vaccine Development and Movement Control. Br J Biomed Sci (2020) 77:168–84. 10.1080/09674845.2020.1826136 32942955

[2] World Health Organisation. Covid-19 Dashboard (2019). https://www.who.int/emergencies/diseases/novel-coronavirus-2019 (Accessed May 12, 2022).

[3] World Health Organisation. World Health Organisation Report on the SARS-CoV-1 Outbreak (2015). https://www.who.int/publications/m/item/summary-of-probable-sars-cases-with-onset-of-illness-from-1-november-2002-to-31-july-2003 (Accessed May 12, 2022).

[4] Zhu N Zhang D Wang W Li X Yang B Song J A Novel Coronavirus from Patients with Pneumonia in China, 2019. N Engl J Med (2020) 382:727–33. 10.1056/NEJMoa2001017 31978945 PMC7092803

[5] Chan JF Kok KH Zhu Z Chu H To KKW Yuan S Genomic Characterization of the 2019 Novel Human-Pathogenic Coronavirus Isolated from a Patient with Atypical Pneumonia after Visiting Wuhan. Emerg Microbes Infect (2020) 9:221–36. 10.1080/22221751.2020.1719902 31987001 PMC7067204

[6] Lu R Zhao X Li J Niu P Yang B Wu H Genomic Characterisation and Epidemiology of 2019 Novel Coronavirus: Implications for Virus Origins and Receptor Binding. Lancet (2020) 395:565–74. 10.1016/S0140-6736(20)30251-8 32007145 PMC7159086

[7] National Library of Medicine. Search for “SARS-Cov-2”. https://www.ncbi.nlm.nih.gov/nuccore/?term=SARS-Cov-2 (Accessed May 12, 2022).

[8] GISAID. https://www.gisaid.org/ (Accessed May 12, 2022).

[9] Zhou P Yang XL Wang XG Hu B Zhang L Zhang W A Pneumonia Outbreak Associated with a New Coronavirus of Probable Bat Origin. Nature (2020) 579:270–3. 10.1038/s41586-020-2012-7 32015507 PMC7095418

[10] Coronaviridae Study Group of the International Committee on Taxonomy of Viruses. The Species Severe Acute Respiratory Syndrome-Related Coronavirus: Classifying 2019-nCoV and Naming it SARS-CoV-2. Nat Microbiol (2020) 5:536–44. 10.1038/s41564-020-0695-z 32123347 PMC7095448

[11] World Health Organisation. Novel Coronavirus (2019-nCoV) SITUATION REPORT - 1 (2020). https://www.who.int/docs/default-source/coronaviruse/situation-reports/20200121-sitrep-1-2019-ncov.pdf?sfvrsn=20a99c10_4 (Accessed May 12, 2022).

[12] World Health Organisation. Coronavirus Disease 2019 (COVID-19) Situation Report – 23 (2020). https://www.who.int/docs/default-source/coronaviruse/situation-reports/20200212-sitrep-23-ncov.pdf?sfvrsn=41e9fb78_4 (Accessed May 12, 2022).

[13] Petersen E Koopmans M Go U Hamer DH Petrosillo N Castelli F Comparing SARS-CoV-2 with SARS-CoV and Influenza Pandemics. Lancet Infect Dis (2020) 20:e238–e244. 10.1016/S1473-3099(20)30484-9 32628905 PMC7333991

[14] Chen N Zhou M Dong X Qu J Gong F Han Y Epidemiological and Clinical Characteristics of 99 Cases of 2019 Novel Coronavirus Pneumonia in Wuhan, China: a Descriptive Study.. Lancet (2020) 395:507–13. 10.1016/S0140-6736(20)30211-7 32007143 PMC7135076

[15] Wang D Hu B Hu C Zhu F Liu X Zhang J Clinical Characteristics of 138 Hospitalized Patients with 2019 Novel Coronavirus-Infected Pneumonia in Wuhan, China.. J Amer Med Assoc (2020) 323:1061–9. 10.1001/jama.2020.1585 PMC7042881 32031570

[16] Yu Y Zhu C Yang L Dong H Wang R Ni H Identification of Risk Factors for Mortality Associated with COVID-19. PeerJ (2020) 8:e9885. 10.7717/peerj.9885 32953279 PMC7473053

[17] Canevelli M Palmieri L Raparelli V Punzo O Donfrancesco C Lo Noce C COVID-19 Mortality Among Migrants Living in Italy. Ann Ist Super Sanità (2020) 56:373–7. 10.4415/ANN_20_03_16 32959804

[18] Rossi PG Marino M Formisano D Characteristics and Outcomes of a Cohort of COVID-19 Patients in the Province of Reggio Emilia. Italy Plos ONE (2020) 15(8):e0238281. 10.1371/journal.pone.0238281 32853230 PMC7451640

[19] Estella A Garcia Garmendia JL de la Fuente C Machado Casas JF Yuste ME Amaya Villar R Predictive Factors of Six-Week Mortality in Critically Ill Patients with SARS-CoV-2: A Multicenter Prospective Study. Med Intensiva (Engl Ed (2021) 46:179–91. 10.1016/j.medin.2021.02.013 PMC7938739 33812670

[20] Pollán M Pérez-Gómez B Pastor-Barriuso R Oteo J Hernan MA Perez-Olmeda M Prevalence of SARS-CoV-2 in Spain (ENE-COVID): a Nationwide, Population-Based Seroepidemiological Study. Lancet (2020) 396:535–44. 10.1016/S0140-6736(20)31483-5 32645347 PMC7336131

[21] Soriano V Barreiro P. Why Such Excess of Mortality for COVID-19 in Spain? Ther Adv Infect Dis (2020) 7:2049936120932755. 10.1177/2049936120932755 32547741 PMC7273618

[22] Fenton NE Neil M Osman M McLachlan S. COVID-19 Infection and Death Rates: the Need to Incorporate Causal Explanations for the Data and Avoid Bias in Testing. J Risk Res (2020) 23:862–5. 10.1080/13669877.2020.1756381

[23] Boin A Lodge M Luesink M. Learning from the COVID-19 Crisis: an Initial Analysis of National Responses. Policy Des Pract (2020) 33:189–204. 10.1080/25741292.2020.1823670

[24] Capano G Howlett M Jarvis DSL Ramesh M Goyal N. Mobilizing Policy (In)Capacity to Fight COVID-19: Understanding Variations in State Responses.. Policy Soc (2020) 39:285–308. 10.1080/14494035.2020.1787628 35039722 PMC8754710

[25] Kapitany-Foveny M Sulyok M. Social Markers of a Pandemic: Modelling the Association between Cultural Norms Land COVID-19 Spread Data. Hum Soc Sci Comms (2020) 7:97. 10.1057/s41599-020-00590-z

[26] Koh D Cunningham AC. Counting Coronavirus Disease 2019 (COVID-19) Cases: Case Definitions, Screened Populations and Testing Techniques Matter. Ann Acad Med Singap (2020) 49:161–5. 10.47102/annals-acadmedsg.202038 32301478

[27] Tsang TK Wu P Lin Y Lau EHY Leung GM Cowling BJ. Effect of Changing Case Definitions for COVID-19 on the Epidemic Curve and Transmission Parameters in mainland China: a Modelling Study. Lancet Public Health (2020) 5:e289–e296. 10.1016/S2468-2667(20)30089-X 32330458 PMC7173814

[28] Jordan RE Adab P Cheng KK. Covid-19: Risk Factors for Severe Disease and Death. Br Med J (2020) 368:m1198. 10.1136/bmj.m1198 32217618

[29] Solomon JJ Heyman B Ko JP Condos R Lynch DA. CT of Post-Acute Lung Complications of COVID-19. Radiology (2021) 301:E383–E395. 10.1148/radiol.2021211396 34374591 PMC8369881

[30] Dillon K Hook C Coupland Z Avery P Taylor H Lockyer A. Pre-hospital Lowest Recorded Oxygen Saturation Independently Predicts Death in Patients with COVID-19. Br Paramed J (2020) 5:59–65. 10.29045/14784726.2020.09.5.3.59 33456398 PMC7783956

[31] Drake TM Docherty AB Harrison EM Quint JK Adamali H Agnew S Outcome of Hospitalization for COVID-19 in Patients with Interstitial Lung Disease. An International Multicenter Study. Am J Respir Crit Care Med (2020) 202(12):1656–65. 10.1164/rccm.202007-2794OC 33007173 PMC7737581

[32] Han X Fan Y Alwalid O Li N Jia X Yuan M Six-month Follow-Up Chest CT Findings after Severe COVID-19 Pneumonia. Radiology (2021) 299:E177–E186. 10.1148/radiol.2021203153 33497317 PMC7841877

[33] Silva Andrade B Siqueira S de Assis Soares WR de Souza Rangel F Santos NO Dos Santos Freitas A Long-COVID and Post-COVID Health Complications: An Up-To-Date Review on Clinical Conditions and Their Possible Molecular Mechanisms. Viruses (2021) 13:700. 10.3390/v13040700 33919537 PMC8072585

[34] Wathen CG Sudlow MF. Pneumonia. Postgrad Med J (1986) 62:396–76. 10.1136/pgmj.62.727.369 3532084 PMC2418683

[35] Ferrara JL Abhyankar S Gilliland DG. Cytokine Storm of Graft Versus-Host Disease: a Critical Effector Role for Interleukin-1. Transpl Proc (1993) 25:1216–7. 8442093

[36] Samson SY Wong KY. The Severe Acute Respiratory Syndrome (SARS). J Neurovirol (2005) 11:455–68. 10.1080/13550280500187724 16287687 PMC7095431

[37] Tisoncik JR Korth MJ Simmons CP Farrar J Martin TR Katze MG. Into the Eye of the Cytokine Storm. Microbiol Mol Biol Rev (2012) 76:16–32. 10.1128/MMBR.05015-11 22390970 PMC3294426

[38] Behrens EM Koretzky GA Review: Cytokine Storm Syndrome: Looking toward the Precision Medicine Era. Arthritis Rheumatol (2017) 69:1135–43. 10.1002/art.40071 28217930

[39] Comer SP Cullivan S Szklanna PB Weiss L Cullen S Kelliher S COVID-19 Induces a Hyperactive Phenotype in Circulating Platelets. Plos Biol (2021) 19:e3001109. 10.1371/journal.pbio.3001109 33596198 PMC7920383

[40] Zhang X Zhang Y Qia W Zhang J Qi Z. Baricitinib, a Drug with Potential Effect to Prevent SARS-COV-2 from Entering Target Cells and Control Cytokine Storm Induced by COVID-19. Int Immunopharmacol (2020) 86:106749. 10.1016/j.intimp.2020.106749 32645632 PMC7328558

[41] Bikdeli B Madhavan MV Jimenez D Chuich T Dreyfus I Driggin E COVID-19 and Thrombotic or Thromboembolic Disease: Implications for Prevention, Antithrombotic Therapy, and Follow-Up: JACC State-Of-The-Art Review.. J Am Coll Cardiol (2020) 75:2950–73. 10.1016/j.jacc.2020.04.031 32311448 PMC7164881

[42] Gerber GF Chaturvedi S How to Recognize and Manage COVID-19-Associated Coagulopathy. Hematol Am Soc Hematol Educ Program (2021) 2021(1):614–20. 10.1182/hematology.2021000297 PMC8791093 34889412

[43] Spyropoulos AC Bonaca MP. Studying the Coagulopathy of COVID-19. Lancet (2022) 399(21):118–9. 10.1016/S0140-6736(21)01906-1 34800425 PMC8598181

[44] Klok FA Kruip MJHA van der Meer NJM Arbous MS Gommers DAMPJ Kant KM Incidence of Thrombotic Complications in Critically Ill ICU Patients with COVID-19. Thromb Res (2020) 191:145–7. 10.1016/j.thromres.2020.04.013 32291094 PMC7146714

[45] Goshua G Pine AB Meizlish ML Chang CH Zhang H Bahel P Endotheliopathy in COVID-19-Associated Coagulopathy: Evidence from a single-centre, Cross-Sectional Study. Lancet Haematol (2020) 7:e575–e582. 10.1016/S2352-3026(20)30216-7 32619411 PMC7326446

[46] Blann AD. Plasma von Willebrand factor, thrombosis, and the endothelium: the first 30 years. Thromb Haemost (2006) 95:49–55. 10.1160/th05-07-0527 16543961

[47] Bikdeli B Madhavan MV Gupta A Jimenez D Burton JR Der Nigoghossian C Pharmacological Agents Targeting Thromboinflammation in COVID-19: Review and Implications for Future Research. Thromb Haemost (2020) 120:1004–24. 10.1055/s-0040-1713152 32473596 PMC7516364

[48] RECOVERY collaborative group. Aspirin in Patients Admitted to Hospital with COVID-19 (RECOVERY): a Randomised, Controlled, Open-Label, Platform Trial. Lancet (2022) 399:143–51. 10.1016/S0140-6736(21)01825-0 34800427 PMC8598213

[49] Clift AK Coupland CAC Keogh RH Diaz-Ordaz K Williamson E Harrison EM Living Risk Prediction Algorithm (QCOVID) for Risk of Hospital Admission and Mortality from Coronavirus 19 in Adults: National Derivation and Validation Cohort Study. BMJ (2020) 371:m3731. 10.1136/bmj.m3731 33082154 PMC7574532

[50] NHS. Coronavirus (COVID-19) Risk Assessment (2021). https://digital.nhs.uk/coronavirus/risk-assessment (Accessed May 12, 2022).

[51] McIntyre PB Aggarwal R Jani I Jawad J Kochhar S MacDonald N COVID-19 Vaccine Strategies Must Focus on Severe Disease and Global Equity. Lancet (2022) 399:406–10. 10.1016/S0140-6736(21)02835-X 34922639 PMC8676417

[52] Oliu-Barton M Pradelski BSR Algan Y Baker MG Binagwaho A Dore GJ Elimination versus Mitigation of SARS-CoV-2 in the Presence of Effective Vaccines. Lancet Glob Health (2022) 10:e142–e147. 10.1016/S2214-109X(21)00494-0 34739862 PMC8563003

[53] Phillips N The Coronavirus Is Here to Stay – Here’s what that Means. Nature (2021) 590:382–4. 10.1038/d41586-021-00396-2 33594289

[54] Murray CJL Piot P. The Potential Future of the COVID-19 Pandemic. Will SARS-CoV-2 Become a Recurrent Seasonal Infection? J Amer Med Assoc (2021) 325:1249–50. 10.1001/jama.2021.2828 33656519

[55] Fricke-Galindo I Falfan-Valencia R. Genetics Insight for COVID-19 Susceptibility and Severity: a Review. Front Immunol (2021) 12:622176. 10.3389/fimmu.2021.622176 33868239 PMC8047200

[56] RECOVERY Collaborative Group. Dexamethasone in Hospitalized Patients with Covid-19. N Engl J Med (2021) 384:693–704. 10.1056/nejmoa2021436 32678530 PMC7383595

[57] RECOVERY Collaborative Group. Tocilizumab in Patients Admitted to Hospital with COVID-19 (RECOVERY). Lancet (2021) 397:1637–45. 33933206 10.1016/S0140-6736(21)00676-0 PMC8084355

[58] Mahase E. Covid-19: Molnupiravir Reduces Risk of Hospital Admission or Death by 50% in Patients at Risk, MSD Reports. Br Med J BMJ (2021) 375:n2422. 10.1136/bmj.n2422 34607801

[59] Reis G Dos Santos Moreira-Silva EA Silva DCM Thabane L Milagres AC Ferreira TS Effect of Early Treatment with Fluvoxamine on Risk of Emergency Care and Hospitalisation Among Patients with COVID-19: the TOGETHER Randomised, Platform Clinical Trial. Lancet Glob Health (2021) 10(21):e4200448–514. 10.1016/S2214-109X(21)00448-4 PMC8550952 34717820

[60] World Health Organisation. The World Health Organisation Document: COVID-19 Clinical Management: Living Guidance (2021). https://www.who.int/publications/i/item/WHO-2019-nCoV-clinical-2021-1 (Accessed May 12, 2022).

[61] The National Institute for Health and Care Excellence (NICE). COVID-19 Rapid Guidelines: Managing COVID-19 (NG191) (2022). https://www.nice.org.uk/search?q=NG191 (Accessed May 12, 2022). 34181371

[62] Couzin-Frankel J. Antiviral Pills Could Change Pandemic’s Course. Science (2021) 374:799–800. 10.1126/science.acx9605 34762459

[63] Kreuzberger N Hirsch C Chai KL Tomlinson E Khosravi Z Popp M SARS-CoV-2-neutralising Monoclonal Antibodies for Treatment of COVID-19. Cochrane Database Syst Rev (2021) 9(9):CD013825. 10.1002/14651858.CD013825.pub2 34473343 PMC8411904

[64] Siemieniuk RA Bartoszko JJ Díaz Martinez JP Kum E Qasim A Zeraatkar D Antibody and Cellular Therapies for Treatment of Covid-19: a Living Systematic Review and Network Meta-Analysis. BMJ (2021) 374:n2231. 10.1136/bmj.n2231 34556486 PMC8459162

[65] Gupta A Gonzalez-Rojas Y Juarez E Crespo Casal M Moya J Rodrigues Falci D Effect of Sotrovimab on Hospitalisation or Death Among High-Risk Patients with Mild to Moderate COVID-19. J Amer Med Assoc (2022) 327:1236–46. 10.1001/jama.2022.2832 PMC8922199 35285853

[66] Gottlieb RL Nirula A Chen P Boscia J Heller B Morris J Effect of Bamlanivimab as Monotherapy or in Combination with Etesevimab on Viral Load in Patients with Mild to Moderate COVID-19: A Randomized Clinical Trial. J Amer Med Assoc (2021) 325:632–44. 10.1001/jama.2021.0202 PMC7821080 33475701

[67] NHS. Treatments for Coronavirus (COVID-19) (2022). https://www.nhs.uk/conditions/coronavirus-covid-19/treatments-for-coronavirus/ (Accessed May 12, 2022).

[68] COVID-19 Vaccine Tracker. https://www.covid-19vaccinetracker.org/authorized-vaccines (Accessed May 12, 2022).

[69] Voysey M Costa Clemens SA Madhi SA Weckx LY Folegatti PM Aley PK Safety and Efficacy of the ChAdOx1 nCoV-19 Vaccine (AZD1222) against SARS-CoV-2: an Interim Analysis of Four Randomised Controlled Trials in Brazil, South Africa, and the UK. Lancet (2021) 397:99–111. 10.1016/S0140-6736(20)32661-1 33306989 PMC7723445

[70] Thomas SJ Moreira ED Jr Kitchin N Absalon J Gurtman A Lockhart S Safety and Efficacy of the BNT162b2 mRNA Covid-19 Vaccine through 6 Months. N Engl J Med (2021) 385:3851761–1773. 10.1056/NEJMoa2110345 PMC8461570 34525277

[71] Baden LR El Sahly HM Essink B Kotloff K Frey S Novak R Efficacy and Safety of the mRNA-1273 SARS-CoV-2 Vaccine. N Engl J Med Overseas Ed (2021) 384:403–16. 10.1056/nejmoa2035389 PMC7787219 33378609

[72] Ghazy RM Ashmawy R Hamdy NA Elhadi YAM Reyad OA Elmalawany D Efficacy and Effectiveness of SARS-CoV-2 Vaccines: A Systematic Review and Meta-Analysis. Vaccines (2022) 10:350. 10.3390/vaccines10030350 35334982 PMC8948677

[73] Huang YZ Kuan CC. Vaccination to Reduce Severe COVID-19 and Mortality in COVID-19 Patients: a Systematic Review and Meta-Analysis. Eur Rev Med Pharmacol Sci (2022) 26:1770–6. 10.26355/eurrev_202203_28248 35302230

[74] Mohammed I Nauman A Paul P Ganesan S Chen KH Jalil SMS The Efficacy and Effectiveness of the COVID-19 Vaccines in Reducing Infection, Severity, Hospitalization, and Mortality: a Systematic Review. Hum Vaccin Immunother (2022) 18:2027160–20. 10.1080/21645515.2022.2027160 35113777 PMC8862168

[75] Altmann DM Boyton RJ. COVID-19 Vaccination: The Road Ahead. Science (2022) 375:1127–32. 10.1126/science.abn1755 35271316

[76] Klamer TA Linschoten M Asselbergs FW. The Benefit of Vaccination against COVID-19 Outweighs the Potential Risk of Myocarditis and Pericarditis. Neth Heart J (2022) 30:190–7. 10.1007/s12471-022-01677-9 35266090 PMC8906525

[77] Iba T Levy JH. Thrombosis and Thrombocytopenia in COVID-19 and after COVID-19 Vaccination. Trends Cardiovasc Med (2022) 32:249–25. 10.1016/j.tcm.2022.02.008 35202800 PMC8861143

[78] Ceacareanu AC Wintrob ZAP. Summary of COVID-19 Vaccine-Related Reports in the Vaccine Adverse Event Reporting System. J Res Pharm Pract (2021) 10:107–13. 10.4103/jrpp.jrpp_49_21 35198503 PMC8809454

[79] Maiese A Baronti A Manetti AC Di Paolo M Turillazzi E Frati P Death after the Administration of COVID-19 Vaccines Approved by EMA: Has a Causal Relationship Been Demonstrated? Vaccines (2022) 10(2):308. 10.3390/vaccines-10020308 35214765 PMC8875435

[80] Marinaki S Adamopolous S Deglannnis D Roussos S Pavlopoulou ID Hatzakis A Immunogenicity of SAVS-CoV-2 BNT162b2 Vaccine in Solid Organ Transplant Recipients. Amer J Transpl (2021) 21:2913–5. 10.1111/ajt.16607 PMC8250574 33864722

[81] Gilbert PB Montefiori DC McDermott A Fong Y Benkeser D Deng W Immune Correlates Analysis of the mRNA-1273 COVID-19 Vaccine Efficacy Clinical Trial.. Science (2022) 375:43–50. 10.1126/science.abm3425 34812653 PMC9017870

[82] Teo SP. Review of COVID-19 Vaccines and Their Evidence in Older Adults. Ann Geriatr Med Res (2021) 25:4–9. 10.4235/agmr.21.0011 33550776 PMC8024166

[83] Flaxman A Marchevsky NG Jenkin D Aboagye J Aley PK Angus B Reactogenicity and Immunogenicity after a Late Second Dose or a Third Dose of ChAdOx1 nCoV-19 in the UK: a Substudy of Two Randomised Controlled Trials (COV001 and COV002).. Lancet (2021) 398:981–90. 10.1016/S0140-6736(21)01699-8 34480858 PMC8409975

[84] Hall VG Ferreira VH Ku T Ierullo M Majchrzak-Kita B Chaparro C Randomized Trial of a Third Dose of mRNA-1273 Vaccine in Transplant Recipients.. N Engl J Med (2021) 385:1244–6. 10.1056/NEJMc2111462 34379917 PMC8385563

[85] Vasileiou E Simpson CR Shi T Kerr S Agrawal U Akbari A Interim Findings from First-Dose Mass COVID-19 Vaccination Roll-Out and COVID-19 Hospital Admissions in Scotland: a National Prospective Cohort Study. Lancet (2021) 397:1646–57. 10.1016/S0140-6736(21)00677-2 33901420 PMC8064669

[86] Agrawal U Katikireddi SV McCowan C Mulholland RH Azcoaga-Lorenzo A Amele S COVID-19 Hospital Admissions and Deaths after BNT162b2 and ChAdOx1 nCoV-19 Vaccinations in 2·57 Million People in Scotland (EAVE II): a Prospective Cohort Study. Lancet Respir Med (2021) 9:1439–49. 10.1016/S2213-2600(21)00380-5 34599903 PMC8480963

[87] Borobia AM Carcas AJ Pérez-Olmeda M Castano L Bertran MJ Garcia-Perez J Immunogenicity and Reactogenicity of BNT162b2 Booster in ChAdOx1-S-Primed Participants (CombiVacS): a Multicentre, Open-Label, Randomised, Controlled, Phase 2 Trial. Lancet (2021) 398:121–30. 10.1016/S0140-6736(21)01420-3 34181880 PMC8233007

[88] UK Coronavirus Dashboard. https://coronavirus.data.gov.uk/ (Accessed April 10, 2022).

[89] Baden LR El Sahly HM Essink B Kotloff K Frey S Novak R Efficacy and Safety of the mRNA-1273 SARS-CoV-2 Vaccine. N Engl J Med Overseas Ed (2020) 384:403–16. 10.1056/NEJMoa2035389 PMC7787219 33378609

[90] Novak N Tordesillas L Cabanillas B. Adverse Rare Events to Vaccines for COVID-19: From Hypersensitivity Reactions to Thrombosis and Thrombocytopenia. Int Rev Immunol (2021) 2021:1–10. 10.1080/08830185.2021.1939696 PMC8290371 34251972

[91] Sokolowska M Thomas Eiwegger T Ollert M Torres MJ Barber D Del Giacco S EAACI Statement on the Diagnosis, Management, and Prevention of Severe Allergic Reactions to COVID-19 Vaccines. Allergy (2021) 76:1629–39. 10.1111/all.14739 33452689 PMC8013422

[92] Shimabukuro T. Allergic Reactions Including Anaphylaxis after Receipt of the First Dose of Moderna COVID-19 Vaccine - United States, December 21, 2020-January 10, 2021. Am J Transpl (2021) 21:1326–31. 10.1111/ajt.16517 PMC8013433 33641268

[93] Arepally GM Ortel TL. Vaccine-induced Immune Thrombotic Thrombocytopenia: what We Know and Do Not Know.. Blood (2021) 138:293–8. 10.1182/blood.2021012152 34323940 PMC8172307

[94] Klok FA Pai M Huisman MV Makris M. Vaccine-induced Immune Thrombotic Thrombocytopenia. Lancet Haematol (2021) -3026(21):e7300306–80. 10.1016/S2352-3026(21)00306-9 PMC8585488 34774202

[95] NICE guideline [NG200]. The National Institute for Health and Care Excellence (NICE) NG200 (2021). https://www.nice.org.uk/guidance/ng200 (Accessed May 12, 2022).

[96] Haas EJ Angulo FJ McLaughlin JM Anis E Singer SR Khan F Impact and Effectiveness of mRNA BNT162b2 Vaccine against SARS-CoV-2 Infections and COVID-19 Cases, Hospitalisations, and Deaths Following a Nationwide Vaccination Campaign in Israel: an Observational Study Using National Surveillance Data. Lancet (2021) 397:1819–29. 10.1016/S0140-6736(21)00947-8 33964222 PMC8099315

[97] NIH Research Matters. Vaccines Prevented up to 140,000 COVID-19 Deaths in U.S (2021). https://www.nih.gov/news-events/nih-research-matters/vaccines-prevented-140000-covid-19-deaths-us (Accessed May 12, 2022).

[98] Public Health England. COVID-19 Vaccine Surveillance Report Week 38 (2021). https://assets.publishing.service.gov.uk/government/uploads/system/uploads/attachment_data/file/1019992/Vaccine_surveillance_report_-_week_38.pdf (Accessed May 12, 2022).

[99] Public Health England. COVID-19 Vaccine Surveillance Report Published (2021). https://www.gov.uk/government/news/covid-19-vaccine-surveillance-report-published (Accessed May 12, 2022).

[100] Antonelli M Penfold RS Merino J Sudre CH Molteni E Berry S Risk Factors and Disease Profile of post-vaccination SARS-CoV-2 Infection in UK Users of the COVID Symptom Study App: a Prospective, Community-Based, Nested, Case-Control Study. Lancet Infect Dis (2022) 22:43–55. 10.1016/S1473-3099(21)00460-6 34480857 PMC8409907

[101] Subbarao K. The success of SARS-CoV-2 Vaccines and Challenges Ahead. Cell Host Microbe (2021) 29:1111–23. 10.1016/j.chom.2021.06.016 34265245 PMC8279572

[102] Huang Y Yang C Xu XF Xu W Liu SW. Structural and Functional Properties of SARS-CoV-2 Spike Protein: Potential Antivirus Drug Development for COVID-19. Acta Pharmacol Sin (2020) 41:1141–9. 10.1038/s41401-020-0485-4 32747721 PMC7396720

[103] Zhang L Jackson CB Mou H Ojha A Peng H Quinlan BD SARS-CoV-2 Spike-Protein D614G Mutation Increases Virion Spike Density and Infectivity. Nat Commun (2020) 11:6013. 10.1038/s41467-020-19808-4 33243994 PMC7693302

[104] Koyama T Weeraratne D Snowdon JL Parida L. Emergence of Drift Variants that May Affect COVID-19 Vaccine Development and Antibody Treatment. Pathogens (2020) 9(5):324. 10.3390/pathogens9050324 PMC7281497 32357545

[105] Korber B Fischer WM Gnanakaran S Yoon H Theiler J Abfalterer W Tracking Changes in SARS-CoV-2 Spike: Evidence that D614G Increases Infectivity of the COVID-19 Virus. Cell (2020) 182(4):812–27. 10.1016/j.cell.2020.06.043 32697968 PMC7332439

[106] Rambaut A Holmes EC O'Toole Á Hill V McCrone JT Ruis C A Dynamic Nomenclature Proposal for SARS-CoV-2 Lineages to Assist Genomic Epidemiology. Nat Microbiol (2020) 5:1403–7. 10.1038/s41564-020-0770-5 32669681 PMC7610519

[107] Department for Business, Energy & Industrial Strategy, Department of Health and Social Care, Government Office for Science, Public Health England, Medical Research Council, and The Rt Hon Alok Sharma MP. UK launches whole genome sequence alliance to map spread of coronavirus (2020). https://www.gov.uk/government/news/uk-launches-whole-genome-sequence-alliance-to-map-spread-of-coronavirus (Accessed May 12, 2022).

[108] Hodcroft EB Zuber M Nadeau S Vaughan TG Crawford KHD Althaus CL Spread of a SARS-CoV-2 Variant through Europe in the Summer of 2020. Nature (2021) 595:707–12. 10.1038/s41586-021-03677-y 34098568

[109] Dubey A Choudhary S Kumar P Tomar S. Emerging SARS-CoV-2 Variants: Genetic Variability and Clinical Implications. Curr Microbiol (2021) 79(1):20. 10.1007/s00284-021-02724-1 34905108 PMC8669229

[110] Raman R Patel KJ Ranjan K. COVID-19: Unmasking Emerging SARS-CoV-2 Variants, Vaccines and Therapeutic Strategies. Biomolecules (2021) 11:993. 10.3390/biom11070993 34356617 PMC8301790

[111] Leung K Shum MH Leung GM Lam TT Wu JT. Early Transmissibility Assessment of the N501Y Mutant Strains of SARS-CoV-2 in the United Kingdom, October to November 2020. Eurosurveillance (2021) 26:2002106. 10.2807/1560-7917.es.2020.26.1.2002106 PMC7791602 33413740

[112] Tao K Tzou PL Nouhin J Gupta RK de Oliveira T Kosakovsky Pond SL The Biological and Clinical Significance of Emerging SARS-CoV-2 Variants. Nat Rev Genet (2021) 22:757–73. 10.1038/s41576-021-00408-x 34535792 PMC8447121

[113] Kirby T. New Variant of SARS-CoV-2 in UK Causes Surge of COVID-19. Lancet Respir Med (2021) 9:e20–e21. 10.1016/S2213-2600(21)00005-9 33417829 PMC7784534

[114] Vohringer HS Sanderson T Sinnott M De Maio N Nguyen T Goater R Genomic Reconstruction of the SARS-CoV-2 Epidemic in England. Nature (2021) 600:506–11. 10.1038/s41586-021-04069-y 34649268 PMC8674138

[115] Tegally H Wilkinson E Giovanetti M Iranzadeh A Fonseca V Giandhari J Detection of a SARS-CoV-2 Variant of Concern in South Africa. Nature (2021) 592:438–43. 10.1038/s41586-021-03402-9 33690265

[116] Faria NR Mellan TA Whittaker C Claro IM Candido DS Mishra S Genomics and Epidemiology of the P.1 SARS-CoV-2 Lineage in Manaus, Brazil. Science (2021) 372:815–21. 10.1126/science.abh2644 33853970 PMC8139423

[117] Public Health England. SARS-CoV-2 Technical Briefing 19 (2021). https://assets.publishing.service.gov.uk/government/uploads/system/uploads/attachment_data/file/1005517/Technical_Briefing_19.pdf (Accessed May 12, 2022).

[118] UK Health Security Agency. SARS-CoV-2 Technical Briefing 32 (2021). https://assets.publishing.service.gov.uk/government/uploads/system/uploads/attachment_data/file/1042688/RA_Technical_Briefing_32_DRAFT_17_December_2021_2021_12_17.pdf (Accessed May 12, 2022).

[119] Cov-Lineages. Pango Lineages: Latest Epidemiological Lineages of SARS-CoV-2 (2021). https://cov-lineages.org/global_report_B.1.617.2.html (Accessed May 12, 2022).

[120] Callaway E Delta Coronavirus Variant: Scientists Brace for Impact. Nature (2021) 595:17–8. 10.1038/d41586-021-01696-3 34158664

[121] Burki TK. Omicron Variant and Booster COVID-19 Vaccines. Lancet Respir Med (2021) 10:e17. 10.1016/S2213-2600(21)00559-2 34929158 PMC8683118

[122] Twohig KA Nyberg T Zaiai A Thelwall S Sinnathamby MA Aliabadi S Hospital Admission and Emergency Care Attendance Risk for SARS-CoV-2 delta (B.1.617.2) Compared with Alpha (B.1.1.7) Variants of Concern: a Cohort Study. Lancet Infect Dis (2022) 22:35–42. 10.1016/S1473-3099(21)00475-8 34461056 PMC8397301

[123] UK Health Security Agency. SARS-CoV-2 Technical Briefing (2021). https://assets.publishing.service.gov.uk/government/uploads/system/uploads/attachment_data/file/1036501/Technical_Briefing_29_published_26_November_2021.pdf (Accessed May 12, 2022).

[124] Chen J Wang R Gilby NB Wei GW. Omicron Variant (B.1.1.529): Infectivity, Vaccine Breakthrough, and Antibody Resistance. J Chem Inf Model (2022) 62:412–22. 10.1021/acs.jcim.1c01451 34989238 PMC8751645

[125] Wang Y Zhang L Li Q Li T Liu S Cui Q The Significant Immune Escape of Pseudotyped SARS-CoV-2 Variant Omicron. Emerg Microbes Infect (2022) 11:1–5. 10.1080/22221751.2021.2017757 34890524 PMC8725892

[126] UK Health Security Agency. SARS-CoV-2 Technical Briefing 30 (2021). https://assets.publishing.service.gov.uk/government/uploads/system/uploads/attachment_data/file/1038404/Technical_Briefing_30.pdf (Accessed May 12, 2022).

[127] Harvey WT Carabelli AM Jackson B Gupta RK Thomson EC Harrison EM SARS-CoV-2 Variants, Spike Mutations and Immune Escape. Nat Rev Microbiol (2021) 19:409–24. 10.1038/s41579-021-00573-0 34075212 PMC8167834

[128] Ito K Piantham C Nishiura H. Relative Instantaneous Reproduction Number of Omicron SARS-CoV-2 Variant with Respect to the Delta Variant in Denmark. J Med Virol (2021) 94:2265–8. 10.1002/jmv.27560 PMC9015237 34967453

[129] UK Health Security Agency. Technical Briefing 33: Underlying Data (2021). https://assets.publishing.service.gov.uk/government/uploads/system/uploads/attachment_data/file/1043695/variants-of-concern-technical-briefing-33-data-england-23-december-2021.ods (Accessed May 12, 2022).

[130] UK Health Security Agency. Omicron Daily Overview (2021). https://assets.publishing.service.gov.uk/government/uploads/system/uploads/attachment_data/file/1044331/20211230_OS__Omicron_Daily_Overview.pdf (Accessed May 12, 2022).

[131] Dejnirattisai W Shaw RH Supasa P Liu C Stuart AS Pollard AJ Reduced Neutralisation of SARS-CoV-2 Omicron B.1.1.529 Variant by post-immunisation Serum. Lancet (2021) 399:234–6. 10.1016/S0140-6736(21)02844-0 34942101 PMC8687667

[132] UK Health Security Agency. SARS-CoV-2 Variants of Concern and Variants under Investigation in England (2021). Technical briefing 31 https://assets.publishing.service.gov.uk/government/uploads/system/uploads/attachment_data/file/1042367/technical_briefing-31-10-december-2021.pdf (Accessed May 12, 2022).

[133] UK Health Security Agency. SARS-CoV-2 Variants of Concern and Variants under Investigation in England (2021). Technical briefing 32: https://assets.publishing.service.gov.uk/government/uploads/system/uploads/attachment_data/file/1042688/RA_Technical_Briefing_32_DRAFT_17_December_2021_2021_12_17.pdf (Accessed May 12, 2022).

[134] UK Health Security Agency. SARS-CoV-2 Variants of Concern and Variants under Investigation in England (2022). Technical briefing 36: https://assets.publishing.service.gov.uk/government/uploads/system/uploads/attachment_data/file/1056487/Technical-Briefing-36-22.02.22.pdf (Accessed May 12, 2022).

[135] UK Health Security Agency. SARS-CoV-2 Variants of Concern and Variants under Investigation in England (2022). Technical briefing 39 https://assets.publishing.service.gov.uk/government/uploads/system/uploads/attachment_data/file/1063424/Tech-Briefing-39-25March2022_FINAL.pdf (Accessed May 12, 2022).

[136] Fonager J Bennedbæk M Bager P Wohlfahrt J Ellegaard KM Ingham AC Molecular Epidemiology of the SARS-CoV-2 Variant Omicron BA.2 Sub-lineage in Denmark, 29 November 2021 to 2 January 2022. Euro Surveill (2022) 27(10):2200181. 10.2807/1560-7917.ES.2022.27.10.2200181 PMC8915403 35272746

[137] Gautret P Hoang VT Jimeno MT Lagier JC Rossi P Fournier PE Severity of the First 207 Infections with the SARS-CoV-2 Omicron BA.2 Variant, in Marseille, France, December, 2021-February, 2022. J Med Virol (2022) 94:3494–7. 10.1002/jmv.27760 35365865 PMC9088598

[138] Bruel T Hadjadj J Maes P Planas D Seve A Staropoli I Serum Neutralization of SARS-CoV-2 Omicron Sublineages BA.1 and BA.2 in Patients Receiving Monoclonal Antibodies.. Nat Med (2022) 28:1297–302. 10.1038/s41591-022-01792-5 35322239

[139] Pedersen RM Bang LL Madsen LW Sydenham TV Johansen IS Jensen TG Serum Neutralization of SARS-CoV-2 Omicron BA.1 and BA.2 after BNT162b2 Booster Vaccination.. Emerg Infect Dis (2022) 28(6):1274–5. 10.3201/eid2806.220503 35356875 PMC9155893

[140] Chen LL Chu AW Zhang RR Hung IFN To KKW. Serum Neutralisation of the SARS-CoV-2 Omicron Sublineage BA.2. Lancet Microbe (2022) 3:e404. 10.1016/S2666-5247(22)00060-X 35373159 PMC8959473

[141] Xia X. Domains and Functions of Spike Protein in Sars-Cov-2 in the Context of Vaccine Design. Viruses (2021) 13:109. 10.3390/v13010109 33466921 PMC7829931

[142] Gómez CE Perdiguero B Esteban M. Emerging SARS-CoV-2 Variants and Impact in Global Vaccination Programs against SARS-CoV-2/covid-19. Vaccines (2021) 9:243. 10.3390/vaccines9030243 33799505 PMC7999234

[143] Noori M Nejadghaderi SA Arshi S Carson-Chahhoud K Ansarin K Kolahi AA Potency of BNT162b2 and mRNA-1273 Vaccine-Induced Neutralizing Antibodies against Severe Acute Respiratory Syndrome-CoV-2 Variants of Concern: A Systematic Review of In Vitro Studies. Rev Med Virol (2021) 32:e2277. 10.1002/rmv.2277 34286893 PMC8420542

[144] Martínez-Flores D Zepeda-Cervantes J Cruz-Reséndiz A Aguirre-Sampieri S Sampieri A Vaca L. SARS-Cov-2 Vaccines Based on the Spike Glycoprotein and Implications of New Viral Variants. Front Immunol (2021) 12:701501. 10.3389/fimmu.2021.701501 34322129 PMC8311925

[145] Tregoning JS Flight KE Higham SL Wang Z Pierce BF. Progress of the COVID-19 Vaccine Effort: Viruses, Vaccines and Variants versus Efficacy, Effectiveness and Escape. Nat Rev Immunol (2021) 21:626–36. 10.1038/s41577-021-00592-1 34373623 PMC8351583

[146] Nathan R Shawa I De La Torre I Pustizzi JM Haustrup N Patel DR A Narrative Review of the Clinical Practicalities of Bamlanivimab and Etesevimab Antibody Therapies for SARS-CoV-2. Infect Dis Ther (2021) 10:1933–47. 10.1007/s40121-021-00515-6 34374951 PMC8353431

[147] Gao Y Cai C Grifoni A Muller TR Niessl J Olofsson A Ancestral SARS-CoV-2-specific T Cells Cross-Recognize the Omicron Variant. Nat Med (2022) 28:472–6. 10.1038/s41591-022-01700-x 35042228 PMC8938268

[148] UK Health Security Agency. SARS-CoV-2 Variants of Concern and Variants Under Investigation in England (2021). Technical Briefing 28. https://assets.publishing.service.gov.uk/government/uploads/system/uploads/attachment_data/file/1033101/Technical_Briefing_28_12_Nov_2021.pdf (Accessed May 12, 2022).

[149] Yelin D Margalit I Yahav D Runold M Bruchfeld J. Long COVID-19 – It’s Not over until? Clin Microbiol Infect (2021) 27:506–8. 10.1016/j.cmi.2020.12.001 33316400 PMC7832095

[150] Korompoki E Gavriatopoulou M Hicklen RS Ntanasis-Stathopoulos I Kastritis E Fotiou D Epidemiology and Organ Specific Sequelae of post-acute COVID19: A Narrative Review. J Infect (2021) 83:1–16. 10.1016/j.jinf.2021.05.004 33992686 PMC8118709

[151] Nalbandian A Sehgal K Gupta A Madhavan MV McGroder C Stevens JS Post-Acute COVID-19 Syndrome. Nat Med (2021) 27:601–15. 10.1038/s41591-021-01283-z 33753937 PMC8893149

[152] NICE NG188. COVID-19 Rapid Guideline: Managing the Long-Term Effects of COVID-19 (2022). https://www.nice.org.uk/guidance/ng188. 33555768

[153] Soriano JB Murthy S Marshall JC Relan P Diaz JV. WHO Clinical Case Definition Working Group on Post-COVID-19 Condition. A Clinical Case Definition of post-COVID-19 Condition by a Delphi Consensus. Lancet Infect Dis (2021) 22:E102–E107. 10.1016/S1473-3099(21)00703-9 34951953 PMC8691845

[154] Jennings G Monaghan A Xue F Mockler D Romero-Ortuno R. A Systematic Review of Persistent Symptoms and Residual Abnormal Functioning Following Acute COVID-19: Ongoing Symptomatic Phase vs. Post-COVID-19 Syndrome. J Clin Med (2021) 10:5913. 10.3390/jcm10245913 34945213 PMC8708187

[155] Research Accessibility Team. The Microvascular Hypothesis Underlying Neurologic Manifestations of Long COVID-19 and Possible Therapeutic Strategies. Cardiovasc Endocrinol Metab (2021) 10:193–203. 10.1097/XCE.0000000000000253 34765889 PMC8575441

[156] Phetsouphanh C Darley DR Wilson DB Howe A Munier CML Patel SK Immunological Dysfunction Persists for 8 Months Following Initial Mild-To-Moderate SARS-CoV-2 Infection. Nat Immunol (2022) 23:210–6. 10.1038/s41590-021-01113-x 35027728

[157] Poudel AN Zhu S Cooper N Roderick P Alwan N Tarrant C Impact of Covid-19 on Health-Related Quality of Life of Patients: A Structured Review. PLoS One (2021) 16:e0259164. 10.1371/journal.pone.0259164 34710173 PMC8553121

[158] Camporota L Cronin JN Busana M Gattinoni L Formenti F. Pathophysiology of Coronavirus-19 Disease Acute Lung Injury. Curr Opin Crit Care (2022) 28(1):9–16. 10.1097/mcc.0000000000000911 34907979 PMC8711311

[159] Ambardar SR Hightower SL Huprikar NA Chung KK Singhal A Collen JF. Post-COVID-19 Pulmonary Fibrosis: Novel Sequelae of the Current Pandemic. J Clin Med (2021) 10:2452. 10.3390/jcm10112452 34205928 PMC8199255

[160] John AE Joseph C Jenkins G Tatler AL. COVID‐19 and Pulmonary Fibrosis: a Potential Role for Lung Epithelial Cells and Fibroblasts. Immunol Rev (2021) 302:228–40. 10.1111/imr.12977 34028807 PMC8237078

[161] Wu X Liu X Zhou Y Yu H Li R Zhan Q 3-month, 6-month, 9-month, and 12-month Respiratory Outcomes in Patients Following COVID-19-Related Hospitalisation: a Prospective Study. Lancet Respir Med (2021) 9:P747–754. 10.1016/S2213-2600(21)00174-0 PMC8099316 33964245

[162] Safont B Tarraso J Rodriguez-Borja E Fernandez-Fabrellas E Sancho-Chust JN Molina V Lung Function, Radiological Findings and Biomarkers of Fibrogenesis in a Cohort of COVID-19 Patients Six Months after Hospital Discharge. Arch Bronconeumol (2021) 58:142–9. 10.1016/j.arbres.2021.08.014 34497426 PMC8414844

[163] Robey RC Kemp K Hayton P Mudawi D Wang R Greaves M Pulmonary Sequelae at 4 Months after COVID-19 Infection: A Single-Centre Experience of a COVID Follow-Up Service. Adv Ther (2021) 38:4505–19. 10.1007/s12325-021-01833-4 34278556 PMC8286847

[164] Ishigami J Kou M Ding N Matsushita K. Cardiovascular Disease and Coronavirus Disease 2019:Epidemiology, Management and Prevention. Curr Epidemiol Rep (2021) 8:1–8. 10.1007/s40471-020-00261-2 33425654 PMC7778411

[165] Tijmes FS Thavendiranathan P Udell JA Seidman MA Hanneman K. Cardiac MRI. Assessment of Nonischemic Myocardial Inflammation: State of the Art Review and Update on Myocarditis Associated with COVID-19 Vaccination. Radiol Cardiothorac Imaging (2021) 3:e210252. 10.1148/ryct.210252 34934954 PMC8686006

[166] He J Zhang B Zhou Q Yang W Xu J Liu T The Prognostic Value of Myocardial Injury in COVID-19 Patients and Associated Characteristics. Immun Inflamm Dis (2021) 9:1358–69. 10.1002/iid3.484 34240818 PMC8427070

[167] Sun S Urbanus RT Ten Cate H de Groot PG de Laat B Heemskerk JWM Platelet Activation Mechanisms and Consequences of Immune Thrombocytopenia. Cells (2021) 10:3386. 10.3390/cells10123386 34943895 PMC8699996

[168] Khandelwal G Ray A Sethi S Harikrishnan HK Khandelwal C Sadasivam B. COVID-19 and Thrombotic Complications – the Role of Anticoagulants, Antiplatelets and Thrombolytics. J Fam Med Prim Care (2021) 10:3561–7. 10.4103/jfmpc.jfmpc_1297_20 PMC8653484 34934647

[169] Roychoudhury S Das A Sengupta P Ahmed ABF Dutta S Choudhury AP Viral Pandemics of the Last Four Decades: Pathophysiology, Health Impacts and Perspectives. Int J Environ Res Public Health (2020) 17(24):9411. 10.3390/ijerph17249411 PMC7765415 33333995

[170] Unwin RJ. The 1918 Influenza Pandemic: Back to the Future? Kidney Blood Press Res (2021) 46:639–46. 10.1159/000519288 34662882

[171] Scarpa R Caso F Costa L Passavanti S Vitale MG Trojaniello C May the Analysis of 1918 Influenza Pandemic Give Hints to Image the Possible Magnitude of corona Virus Disease-2019 (COVID-19)? J Transl Med (2020) 18:489. 10.1186/s12967-020-02673-6 33353549 PMC7753514

[172] GBD 2016 Causes of Death Collaborators. Global, Regional and National Age-Sex Specific Mortality for 264 Causes of Death 1980-2016: a Systematic Analysis for the Global burden of Disease Study 2016. Lancet (2017) 390:1151–210. 10.1016/S0140-6736(17)32152-9 28919116 PMC5605883

[173] UK Office for National Statistics. UK Office for National Statistics Figures on Deaths Due to Specific Causes (2022). https://www.nomisweb.co.uk/home/Search?context=&term=mortality (Accessed May 12, 2022).

[174] Strongman H Carreira H De Stavola BL Bhaskaran K Leon DA. Factors Associated with Excess All-Cause Mortality in the First Wave of the COVID-19 Pandemic in the UK: A Time Series Analysis Using the Clinical Practice Research Datalink.. PLOS Med (2022) 19(1):e1003870. 10.1371/journal.pmed.1003870 34990450 PMC8735664

[175] Office for National Statistics. Deaths of Those Under 60 From COVID-19 With No Co-Morbidities and Mortality Rates in 2020 (2021). https://www.ons.gov.uk/aboutus/transparencyandgovernance/freedomofinformationfoi/deathsofthoseunder60fromcovid19withnocomorbiditiesandmortalityratesin2020 (Accessed May 12, 2022).

[176] Oboza P Ogarek N Olszanecka-Glinianowicz M Kocelak P. COVID-19 and Obesity: the Confrontation of Two Pandemics. Eur Rev Med Pharmacol Sci (2022) 26:695–709. 10.26355/eurrev_202201_27896 35113445

[177] Yates T Summerfield A Razieh C Banerjee A Chudasama Y Davies MJ A Population-Based Cohort Study of Obesity, Ethnicity and COVID-19 Mortality in 12.6 Million Adults in England.. Nat Commun (2022) 13(1):624. 10.1038/s41467-022-28248-1 35110546 PMC8810846

[178] Office for National Statistics. Deaths registered weekly in England and Wales, provisional (2022). https://www.ons.gov.uk/peoplepopulationandcommunity/birthsdeathsandmarriages/deaths/datasets/weeklyprovisionalfiguresondeathsregisteredinenglandandwales

[179] Castiello T Georgiopoulos G Finocchiaro G Claudia M Gianatti A Delialis D COVID-19 and Myocarditis: a Systematic Review and Overview of Current Challenges. Heart Fail Rev (2022) 27:251–61. 10.1007/s10741-021-10087-9 33761041 PMC7988375

[180] Han Q Zheng B Daines L Sheikh A. Long Term Sequelae of COVID-19: A Systematic Review and Meta-Analysis of One-Year Follow-Up Studies on post-COVID Symptoms. Pathogens (2022) 11(2):269. 10.3390/pathogens11020269 35215212 PMC8875269

[181] UK Health Security Agency. Weekly National Influenza and COVID-19 Surveillance Report Week 2 Report (Up to Week 1 Data) (2022). https://assets.publishing.service.gov.uk/government/uploads/system/uploads/attachment_data/file/1046284/weekly-flu-and-covid-19-report-week-2-2022.pdf (Accessed May 12, 2022).

[182] Stout S Murphy H Pandya A Yeh HW Portnoy J. The Effect of Coronavirus Disease 2019 on Asthma Visits.. Ann Allergy Asthma Immunol (2022) 128(22):59400048–5955. 10.1016/j.anai.2022.01.027 PMC8801266 35101645

[183] NHS Digital. National Diabetes Audit Programme (2011). https://digital.nhs.uk/data-and-information/clinical-audits-and-registries/national-diabetes-audit (Accessed May 12, 2022).

[184] Dupraz J Le Pogam MA Peytremann-Bridevaux I Early Impact of the COVID-19 Pandemic on In-Person Outpatient Care Utilisation: a Rapid Review. BMJ Open (2022) 12(3):e056086. 10.1136/bmjopen-2021-056086 PMC8895419 35241471

[185] Bakhshandeh B Jahanafrooz Z Abbasi A Goli MB Sadeghi M Mottaqi MS Mutations in SARS-CoV-2; Consequences in Structure, Function, and Pathogenicity of the Virus. Microb Pathog (2021) 154:104831. 10.1016/j.micpath.2021.104831 33727169 PMC7955574

[186] Wabalo EK Dubiwak AD Senbetu MW Gizaw TS. Effect of Genomic and Amino Acid Sequence Mutation on Virulence and Therapeutic Target of Severe Acute Respiratory Syndrome Coronavirus-2 (SARS COV-2). Infect Drug Resist (2021) 14:2187–92. 10.2147/IDR.S307374 34163183 PMC8214021

[187] Hui KPY Ho JCW Cheung MC Ng KC Ching RHH Lai KL SARS-CoV-2 Omicron Variant Replication in Human Bronchus and Lung Ex Vivo. Nature (2022) 603:715–20. 10.1038/s41586-022-04479-6 35104836

[188] Iacobucci G. Covid-19: Government Plans to Remove All Remaining Restrictions in England a Month Early. BMJ (2022) 376:o355. 10.1136/bmj.o355 35140071

[189] Office for National Statistics. Coronavirus (COVID-19) Infection Survey technical article Coronavirus (COVID-19) Infection Survey technical article: waves and lags of COVID-19 in England, June 2021 (2021). https://www.ons.gov.uk/peoplepopulationandcommunity/healthandsocialcare/conditionsanddiseases/articles/coronaviruscovid19infectionsurveytechnicalarticle/wavesandlagsofcovid19inenglandjune2021

[190] Schultze A Nightingale E Evans D Hulme W Rosello A Bates C Mortality Among Care Home Residents in England during the First and Second Waves of the COVID-19 Pandemic: an Observational Study of 4.3 Million Adults over the Age of 65. The Lancet Reg Health - Europe (2022) 14:100295. 10.1016/j.lanepe.2021.100295 PMC8743167 35036983

[191] Mirzaei R Mahdavi F Badrzdeh F Hosseini-Fard SR Heidary M Jeda AS The Emerging Role of microRNAs in the Severe Acute Respiratory Syndrome Coronavirus 2 (SARS-CoV-2) Infection.. Int Immunopharmacol (2021) 90:107204. 10.1016/j.intimp.2020.107204 33221169 PMC7664359

[192] Bautista-Becerril B Pérez-Dimas G Sommerhalder-Nava PC miRNAs, from Evolutionary Junk to Possible Prognostic Markers and Therapeutic Targets in COVID-19. Viruses (2021) 14(1):41. 10.3390/v14010041 35062245 PMC8781105

[193] Strongman H Carreira H De Stavola BL Bhaskaran K Leon DA. Factors Associated with Excess All-Cause Mortality in the First Wave of the COVID-19 Pandemic in the UK: A Time Series Analysis Using the Clinical Practice Research Datalink. Plos Med (2022) 19(1):e1003870. 10.1371/journal.pmed.1003870 34990450 PMC8735664

[194] Mahase E. Covid-19: Outbreak Could Last until spring 2021 and see7.9 Million Hospitalised in the UK. BMJ (2020) 368:m1071. 10.1136/bmj.m1071 32179567

[195] Mahase E. Covid-19: UK Starts Social Distancing after New Model Points to 260 000 Potential Deaths. BMJ (2020) 368:m1089. 10.1136/bmj.m1089 32184205

[196] del Rio C Malani PN. COVID-19 in 2020 – The Beginning of the End or the End of the Beginning? J Amer Med Assoc (2022) 327(24):2389–90. 10.1001/jama.2022.9655 35622357

[197] UK Health Security Agency. SARS-CoV-2 Variants of Concern and Variants Under Investigation. Technical Briefing 43. Available at: https://assets.publishing.service.gov.uk/government/uploads/system/uploads/attachment_data/file/1085404/Technical-Briefing-43.pdf (Accessed June 28, 2022)

